# Diverse Enzymes With Industrial Applications in Four Thraustochytrid Genera

**DOI:** 10.3389/fmicb.2020.573907

**Published:** 2020-10-20

**Authors:** Hsiu-Chin Lin, Wei-Hao Li, Chi-Chih Chen, Tien-Hsing Cheng, Yu-Hsuan Lan, Ming-Der Huang, Wen-Ming Chen, Jo-Shu Chang, Hsin-Yang Chang

**Affiliations:** ^1^Department of Marine Biotechnology and Resources, National Sun Yat-sen University, Kaohsiung, Taiwan; ^2^Doctoral Degree Program in Marine Biotechnology, National Sun Yat-sen University, Kaohsiung, Taiwan; ^3^Department of Biological Sciences, National Sun Yat-sen University, Kaohsiung, Taiwan; ^4^Department of Seafood Science, National Kaohsiung University of Science and Technology, Kaohsiung, Taiwan; ^5^Department of Chemical and Materials Engineering, College of Engineering, Tunghai University, Taichung, Taiwan; ^6^Research Center for Smart Sustainable Circular Economy, Tunghai University, Taichung, Taiwan; ^7^Department of Chemical Engineering, National Cheng Kung University, Tainan, Taiwan; ^8^Department of Life Sciences and Institute of Genome Sciences, National Yang-Ming University, Taipei, Taiwan

**Keywords:** thraustochytrids, transcriptome, industrial enzyme, L-asparaginase, phytase, urease

## Abstract

Thraustochytrids are heterotrophic fungus-like protists that can dissolve organic matters with enzymes. Four strains, AP45, ASP1, ASP2, and ASP4, were isolated from the coastal water of Taiwan, and respectively identified as *Aurantiochytrium* sp., *Schizochytrium* sp., *Parietichytrium* sp., and *Botryochytrium* sp. based on 18S rRNA sequences. Transcriptome datasets of these four strains at days 3-5 were generated using Next Generation Sequencing technology, and screened for enzymes with potential industrial applications. Functional annotations based on KEGG database suggest that many unigenes of all four strains were related to the pathways of industrial enzymes. Most of all four strains contained homologous genes for 15 out of the 17 targeted enzymes, and had extra- and/or intra-cellular enzymatic activities, including urease, asparaginase, lipase, glucosidase, alkaline phosphatase and protease. Complete amino sequences of the first-time identified L-asparaginase and phytase in thraustochytrids were retrieved, and respectively categorized to the Type I and BPPhy families based on phylogenetic relationships, protein structural modeling and active sites. Milligram quantities of highly purified, soluble protein of urease and L-asparaginase were successfully harvested and analyzed for recombinant enzymatic activities. These analytical results highlight the diverse enzymes for wide-range applications in thraustochytrids.

## Introduction

Thraustochytrids (Kingdom Chromista: Superphylum Heterokonta: Phylum Bygira: Class Labyrinthulea: Order Thraustochytrida: Family Thraustochytriidae) are fungus-like protists categorized into at least ten genera and 40 species (Honda et al., 1999; Dellero et al., 2018a; Marchan et al., 2018; Morabito et al., 2019). Several thraustochytrid strains have been successfully cultured and applied to produce high-value functional compounds. The most prominent products of thraustochytrids are omega-3 fatty acids, mostly Docosahexaenoic Acid (DHA) (Lewis et al., 1999; Lee Chang et al., 2014). DHA plays significant roles in many aspects of animal growth and development, such as cell signaling, cell membrane fluidity, and improvement of inflammation response systems and learning (Horrocks and Yeo, 1999; Colomer et al., 2007; Aasen et al., 2016). Thraustochytrids thus provide a sustainable substitute for wild fish and krill, which are current commercial sources of DHA. Other promising high-value products of thraustochytrids are carotenoids (Aki et al., 2003; Aasen et al., 2016; Park et al., 2018), including β-carotene, astaxanthin, canthaxanthin, pheonicoxanthin, and echinenone. Astaxanthin is particularly valuable, because of its highly valued human health benefits (reviewed by Higuera-Ciapara et al., 2006). Carotenoids are often adopted in food, cosmetics and animal feed, due to their coloring and antioxidative functions (reviewed by Mezzomo and Ferreira, 2016).

Heterotrophic thraustochytrids have been reported to process dissolved organic matters with enzymes better than the closely related autotrophic microalgae (Marchan et al., 2018). These degradative macromolecules include polysaccharide hydrolase, protease and urease (Bremer and Talbot, 1995; Raghukumar, 2008; Taoka et al., 2009). For example, alkaline lipases generated by *Thraustochytrium* sp. can hydrolyze long chain triglycerides, and thus have wide applications in the detergent, cosmetic and food industries (Hasan et al., 2006; Kanchana et al., 2011). Cellulose is critical for producing ethanol with low cost and high yield, as a biofuel in order to save the urgent energy crisis and climate change issues. Interestingly, heterologous expression of cellobiohydrolase type I gene (cbh1) from thraustochytrid *Schizochytrium aggregatum* in yeast can enhance cellulose digestion and ethanol production (Brevnova et al., 2013). Taoka et al. (2009) also examined the extracellular enzymes produced by thraustochytrids, and suggested that these marine protists may play a significant role in nitrogen and carbon cycles in the marine ecosystem.

Transcriptome datasets of several thraustochytrid strains have been generated and applied to investigate their functional profiles. DHA is the most promising product of thraustochytrids. Ma et al. (2015) screened the transcriptome dataset of *Aurantiochytrium* sp. SD11 for enzymes involved in the production of unsaturated fatty acid at low temperature. Additionally, the lipid metabolism profile can change with life cycle stages, as reported from a congeneric transcriptome dataset (Dellero et al., 2020). In order to increase the yield of DHA in *Schizochytrium* sp. S056, Chen et al. (2016) replaced glucose with glycerol as the carbon source, and the transcriptome results indicated that the enhancement of fatty acid synthase (FAS) system led to the accumulation of DHA. In contrast to the merits of DHA, thraustochytrids can also be harmful to humans. Rubin et al. (2014) performed transcriptome deep-sequencing to Quahog Parasite Unknown (QPX) thraustochytrid, which is parasitic and infectious to hard clams *Mercenaria mercenaria*, valuable seafood products in the United States. They found putative virulence-related molecules, including peptidases, antioxidant enzymes and proteins, expressed in high disease prevalence condition.

This study aimed to identify 17 enzymes with potential industrial applications, including alkaline phosphatase, carboxylesterase, chitinase, lipase, and glycohydrolase, by screening *de novo* transcriptome datasets and assessing enzymatic activities in thraustochytrids. A heterologous *Escherichia coli* expression system was also used to obtain recombinant urease, L-asparaginase and phytase for further characterization and biological activity evaluation. Thraustochytrid samples were collected from the coastal water of Taiwan, and four strains belonging to different genera were isolated, namely AP45, ASP1, ASP2, and ASP4. This sampling of strains with wide taxonomic coverage should well reveal the diversity of industrial enzymes in thraustochytrids.

## Materials and Methods

### Isolation of Thraustochytrids

Sediment samples were collected from the mangrove area of Qigu, Taiwan. The samples were filtered through a 100 mm filter net, followed by a 10 mm filter net. 200 μl filtrate was spread on a Glucose-Yeast Extract-Peptone (GYP) medium plate comprising 10 g l^–1^ glucose, 0.5 g l^–1^ yeast extract, 1 g l^–1^ mycological peptone, 15 g l^–1^ agar, 0.1 g l^–1^ Ampicillin, 0.1 g l^–1^ Kanamycin, 0.1 g l^–1^ Streptomycin, and 15 g l^–1^ natural seawater (Red Sea Salt, Red Sea, Houston, TX, United States) and incubated at 28°C for 14 days. Each thraustochytrid colony was confirmed based on morphology under microscope, and sub-cultured on a new plate for another 14 days. Four strains, AP45, ASP1, ASP2, and ASP4, with distinguishable taxonomic characteristics were selected and further cultivated in a 25 ml tube with autoclaved GYP medium, and incubated at 150 rpm and 28°C. The cell growth rate revealed by optical density value at 600 nm (OD600) suggested that day 3 and day 5 individually represents the middle stage of the exponential growth phase and the stationary phase in AP45, while the late stage of exponential growth phase and the stationary phase in ASP4. Both ASP1 and ASP2 exhibited a relatively low growth rate, reaching the stationary phase in days 3–5. Significant color change was observed during the same period in AP45 and ASP2, suggesting a change in the gene expression profile. Transcriptome datasets of these two days from strains with different growth ability should cover a wide range of genes related to enzymes expressed in varying physiological status. Therefore 5 ml sample with OD600 of 0.6 were obtained for RNA extraction on days 3 and 5 (except days 3, 4, 5, and 6 for AP45) for each strain, respectively.

### DNA Extraction, Sequencing and Taxonomic Assignment

Total genomic DNA of each strain was extracted using Genomic DNA Mini Kit (Plant) (Geneaid, New Taipei City, Taiwan) following the manufacturer’s instructions. Partial sequences of nuclear gene 18S rRNA were amplified by polymerase chain reaction (PCR) with two primer sets: SR01 and SR05, 586 and 1286 (Nakayama et al., 1996; Hadziavdic et al., 2014). The PCR solution contained 40 ng of template DNA, 5 μl Taq DNA Polymerase Master Mix (1.5 mM MgCl_2_; Ampliqon, Denmark), 1 μM of each primer, and ddH_2_O with a final volume of 10 μl. The reaction was performed under the following conditions: 90 s at 95°C for initial denaturing, 35 cycles of 30 s at 95°C, 15 s at 53–65°C, 50 s at 72°C with a final extension for 5 min at 72°C. The amplified fragments were then sub-cloned into a pET21 vector and sequenced by an ABI-Prism automated DNA sequencer (by Genomics, Taipei, Taiwan). The sequences of each sample were assembled and edited in Geneious 8.1.8^1^. For preliminary species identification, BLAST search using the retrieved 18S rRNA sequence as query was performed on the GenBank database of the National Center for Biotechnology Information. The blast results indicated that all four strains were closely related to the samples collected from Seto Inland Sea, Japan published by Ueda et al. (2015). Therefore, an 18S rRNA data matrix was built with the four strains from this study and 191 isolates from Ueda et al. (2015). Sequences were aligned with CLUSTAL X (Thompson et al., 1997) using default settings, adjusted by eye in Geneious 8.1.8, then trimmed following Dellero et al. (2018a). The best substitution model was selected with corrected Akaike Information Criterion (AICc) and applied for phylogenetic reconstruction using the Maximum-Likelihood (ML) method implemented in MEGA X (Kumar et al., 2018). Node support was estimated with 5,000 bootstrap replicates (Felsenstein, 1985). Taxonomic assignment of each strain was determined by both the phylogenetic relationships and pairwise sequence identity (>97%: conspecific, 97%-92%: congeneric) proposed by Dellero et al. (2018a).

### RNA Extraction, cDNA Library Preparation, and Sequencing

Total RNA of each sample was extracted using TRIzol^§^ Reagent (Invitrogen, Camarillo, CA) following manufacturer’s protocol. RNA quality assessment was performed with a Bioanalyzer 2100 with RNA 6000 labchip kit (Agilent Technologies, Santa Clara, CA, United States). The mRNA was enriched from total RNA using oligo(dT) beads, fragmented randomly, then reverse transcribed into cDNA using random hexamers. The cDNA library of each sample was prepared by Illumina TruSeq RNA Sample Prep Kits v2, and subsequently sequenced with HiSeq 2500 High-Throughput Mode v4 with paired-end 125 base-pair reads operated by Novogene Co., Ltd., according to the manufacturer’s instructions (Illumina, San Diego, CA).

### Sequence *de novo* Assembly, Annotation, and Bioinformatics Analyses

RNA reads from different days of culture in the same strain were combined into a single dataset in order to obtain a consolidated set of contigs. The sequence read quality was checked with FastQC v0.11.5 (Babraham Bioinformatics, Cambridge, United Kingdom) and filtered using the Trimmomatic v0.35 (Bolger et al., 2014) to discard adaptor sequences and low-quality reads. The trimmed reads were then applied to do *de novo* assemblies with Trinity v2.2.0 (Grabherr et al., 2011; Haas et al., 2013). Corset (Davidson and Oshlack, 2014) was adopted to cluster the assembled contigs based on shared reads and filter out contigs with less than 10 reads. The longest transcripts of each cluster were selected as unigenes, which were then annotated and applied for the following analyses. Gene functional annotation was performed at the public databases of Nr (NCBI non-redundant protein sequences), Nt (NCBI nucleotide sequences), Pfam (Protein family), COG (Cluster of Orthologous Groups of proteins) and KOG (euKaryotic Orthologous Groups), Swiss-Prot, KEGG (Kyoto Encyclopedia of Genes and Genome), and GO (Gene Ontology). The *E*-value threshold at Nr, Nt, Swiss-Prot were set to 1 × 10^–5^, and KOG to 1 × 10^–3^. The *E*-value threshold at Pfam, GO, KEGG were set to 0.01, 1 × 10^–6^ and 1 × 10^–10^, respectively. CDS (coding sequence) was predicted and extracted from unigene sequences based on the BLAST results of Nr and Swiss-Prot databases. v3.0.3 (Iseli et al., 1999) was adopted to predict CDS of unigenes with no hits in BLAST.

### Screening of Industrial Enzymes

Amino acid sequences of 17 enzymes, namely amylase, alkaline phosphatase, carboxylesterase, cellulase, chitinase, dioxygenase, α-glucosidase, β-glucosidase, ketosynthase, laccase, L-asparaginase, lipase, monooxygenase, peroxidase, phytase, protease, and urease, were downloaded from the GenBank database (Supplementary Table 1). These sequences were used as the query to search homologs from the transcriptome dataset of the four strains with *E* = 10^–5^ as the cutoff point by BLAST + 2.9.0. For L-asparaginase and phytase which complete amino acid sequences of thraustochytrid homologs were retrieved, phylogenetic relationships were further reconstructed as described above. Besides the thraustochytrid sequences, the phylogenetic data matrix also included homologs from RCSB PDB (Protein Data Bank) and previous publications. Amino acid sequences were aligned, and the conserved sites were predicted by InterPro (Hunter et al., 2009).

### Enzymatic Activity Assays

Enzymatic activity was analyzed on the fifth day after inoculation. The extracellular enzymes excreted out in liquid medium were separated from cells by centrifuge at 7,000 rpm for 5 min at 4°C. For the intracellular enzymes, the harvested cells of four selected strains were then suspended in Tris–HCl buffer at pH 7.0. After mild centrifugation, the resulting pellet was washed twice in the same buffer, and lysed via ultrasonication process in the presence of protease inhibitors (Roche, cOmplet, EDTA-free Protease Inhibitor Cocktail). Sonication was used to disrupt the cell suspension with 7 burst of 10 s followed by intervals of 20 s for cooling on ice. To obtain crude extracts, the lysates were centrifuged at 13,000 × *g* for 10 min at 4°C, and the supernatant was collected for activity analysis. Enzymatic assay was determined by using colorimetric and fluorometric assay kit (BioVision, Thermo Fisher and Abcam), and performed in 96-well microplates, which were detected with the BioTec Epoch^2^ Microplate Spectrophotometer.

The urease assay was performed according to the standard method described in protocol of BioVision Assay Kit (catalog no. K378). Each reaction was incubated and added into the wells of a 96-well microplate at 37°C for 5 to 30 min. The released ammonia was measured at 670 nm, and quantified based on the ammonia standard curve. For the L-asparaginase activity assay (Biovision, catalog no. K754), L-asparagine was hydrolyzed by enzyme to L-aspartate, which is converted to pyruvate forming a stable chromophore with a colorless probe. Each reaction was incubated at 25°C for 10 to 30 min, and the released chromophore molecules were measured at 570 nm. Phytase activity was measured according to the method described in the previous report (Bae et al., 1999). The amount of released inorganic phosphate was detected at 700 nm using sodium phytate as the substrate. Each reaction was incubated at 37°C for 10 to 30 min. For the lipase activity assay (Biovision, catalog no. K722), the enzyme hydrolyzed a triglyceride molecule to form OxiRed probe (peroxidase fluorogenic substrate) linked glycerol, which was measured at 570 nm. Reactions were incubated at 37°C for 10 to 50 min for activity detection. The α-glucosidase activity (Biovision, catalog no. K690) was detected as the amount of p-nitrophenol, measured colorimetrically at 410 nm after the sample incubation at 25°C for 10 to 50 min. The alkaline phosphatase activity (Biovision, catalog no. K412) can be detected as substrate p-nitrophenyl phosphate (pNPP) dephosphorylated. The samples of the final reaction turned yellow, which was measured colorimetrically at 405 nm after the sample incubation at 25°C for 10 to 50 min. The protease activity (Thermo Fisher, catalog no. 23263) can be detected at 410 nm as any enzyme cleaves casein substrate into peptide fragments after incubating at 25°C for 20 min. For the amylase activity assay (Biovision, catalog no. K711), enzyme cleaves a substrate ethylidene-pNP-G7 and results in a colorimetric product, which can be detected at 405 nm. Reactions were incubated at 25°C for 10 to 30 min for activity assay. The cellulase activity (Abcam, catalog no. 189817) was detected as substrate resorufin-cellobioside cleavage. The released fluorescent resorufin was measured at range Ex/Em = 550/595 nm (peak Ex/Em = 571/585 nm) in a fluorescence spectrophotometer. The reactions were incubated at 25°C for 10 to 50 min. Experiments were assayed with protease inhibitors (exclusive of the protease activity) in triplicate and performed three times.

For the recombinant enzyme assay (urease and L-asparaginase), the reaction mixture (200 μl) contains 50 mM Tris-HCl at pH 7.0, 150 mM NaCl, substrates and 2–5 μg purified enzyme. Each reaction was incubated and added into the wells of a 96-well microplate as previous description. The released product was measured colorimetrically and quantified based on the standard curve. The activity was expressed in units of μmol mg^–1^min^–1^ (U/mg), and all reactions were performed in triplicate.

### Protein Expression and Purification

Full-length open reading frames (ORFs) corresponding to L-asparaginase (ASP1), phytase (ASP1) and urease (AP45 and ASP4) were subcloned into a pET21 vector, modified to incorporate a tobacco etch virus (TEV) protease cleavage site between an N-terminal 6His-tag and the polylinker. After confirming constructs by DNA sequencing, each proposed enzyme was expressed in *E. coli* BL21 (DE3). Cells were first grown at 37°C until they reached an OD_600_ of ∼0.6, at which point the culture was induced with 0.5 mM isopropyl β-D-1-thiogalactopyranoside (IPTG), and cultivated further at 20°C for 20 h. For protein purification, the cells were harvested and resuspended in buffer A (50 mM Tris, pH 7.5, 500 mM NaCl), and then disrupted in the presence of protease inhibitors using a high-pressure microfluidizer. After clearing by centrifugation, the supernatant was batch purified by Ni-NTA affinity chromatography. This strategy typically resulted in milligram quantities of highly purified, soluble protein for L-asparaginase and urease, but not phytase in this study. Protein concentration was estimated by A280 using derived extinction coefficients. About 200 μl aliquots of the purified protein solution were flash frozen in liquid nitrogen, and stored at -80°C.

### Construction of the ASP1 Phytase Structural Model

The structural model for ASP1 phytase was built using homology modeling procedures based on the known three-dimensional structure of phytase from *Bacillus amyloliquefaciens* (Protein Data Bank ID: 1CVM, 1H6L, 1POO, 1QLG, and 2POO). The 3D structural models for ASP1 phytase and further energy minimization procedures were built with the Discovery Studio 2.5 software from Accelrys (San Diego, CA, United States). Finally, five candidates were selected, and the best model was chosen and optimized from these. Data were plotted by PyMol software (Schrödinger, New York, United States).

## Results

### Taxonomic Assignment

**FIGURE 1:**
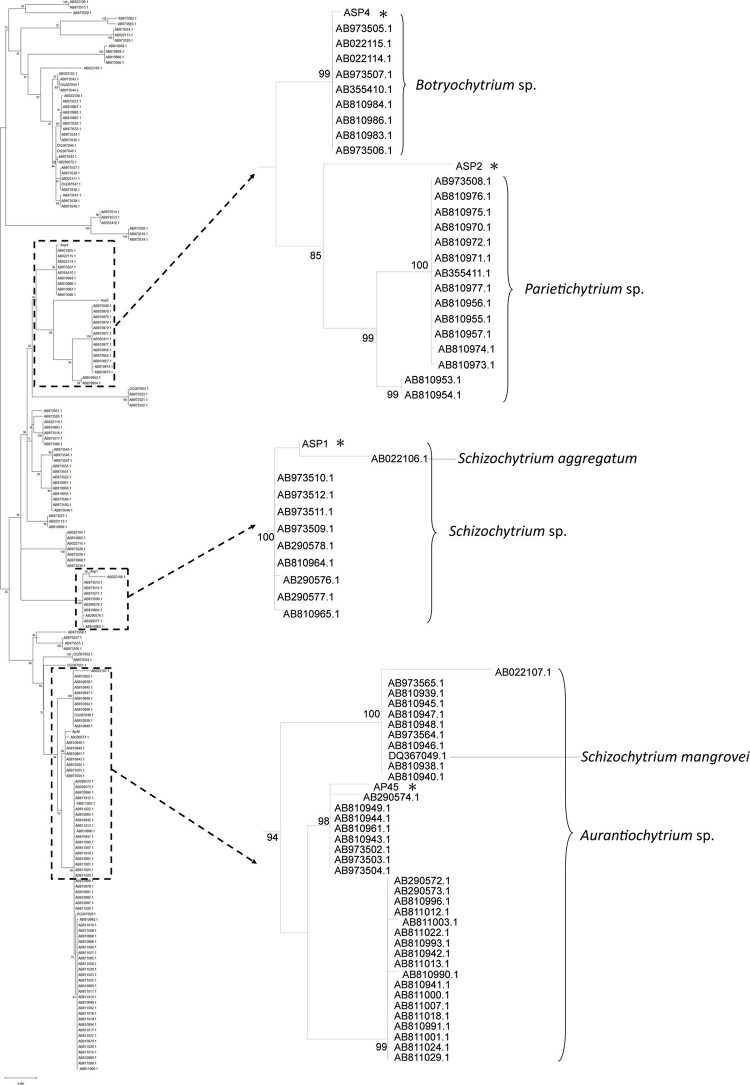
Maximum-Likelihood (ML) tree based on 18S rRNA sequences of the four thraustochytrid strains isolated from this study, and 191 isolates from Ueda et al. (2015). Node labels are bootstrap values (%) calculated with 5,000 replicate searches, values below 75 are not shown. Scale bars indicate the numbers of expected substitution per site with GTR + G + I nucleotide substitution model.

18S rRNA sequences of the four selected strains were retrieved with the total length ranged from 1,199 to 1,718 bp (GenBank Accession Number MK108007, 108006, 108002, and 108005). The length of trimmed data matrix was 1,320 bp. General Time Reversible Model plus gamma-distributed rates among sites and proportion of invariant sites (GTR + G + I) was selected as the best nucleotide substitution model. In the phylogenetic tree, AP45, ASP1, and ASP4 were nested in the sequences of *Aurantiochytrium* sp., *Schizochytrium* sp., and *Botryochytrium* sp., respectively (Figure 1). The maximum pairwise sequence identity of each pair reached the congeneric level (AP45 and *Aurantiochytrium* sp. AB810944.1: 95.79%, ASP1 and *Schizochytrium* sp. AB973512.1: 94.13%, ASP4 and *Botryochytrium* sp. AB810984.1: 93.80%). The maximum pairwise sequence identity of ASP2 was 80.96% with *Parietichytrium* sp. AB810953.1 and ASP2 was sister to two *Parietichytrium* spp. with strong node support (bootstrap value = 85%).

### Identification of Expressed Transcripts in the Transcriptome Datasets

The Illumina deep sequencing generated approximately 28 × 10^6^ to 57 × 10^6^ raw reads for each samples (Table 1) and were submitted to GenBank (Bioproject accession number PRJNA648776). After removing adaptors and low quality reads, the clean bases ranged from 4.2G to 8G (Table 1). The total numbers of assembled transcripts for each of the four selected strains were from 19,763 to 49,729, average length from 1,051 bp to 1,888 bp, maximum length from 19,081 bp to 33,102 bp, and total number of unigenes from 19,745 to 49,677 (Table 2). In total, 61.79% (AP45), 62.3% (ASP1), 64.72% (ASP2), and 73.51% (ASP4) unigenes were successfully annotated in at least one database, while only 5.54% (AP45), 4.58% (ASP1), 2.44% (ASP2), and 2.64% (ASP4) were annotated from all seven public databases (Supplementary Table 2). The *E*-value and score distribution of the best hits in the Nr database show that 26.48% (AP45), 22.85% (ASP1), 31.15% (ASP2), and 28.32% (ASP4) of the matched sequences were highly homologous, with score >500, and 24.97% (AP45), 21.09% (ASP1), 29.08% (ASP2), and 26.50% (ASP4) with *E* < 1 × 10^–50^.

**TABLE 1:** Summary of raw reads of samples by Illumina deep sequencing.

Strain name	Day	Raw Reads	Clean Reads	Clean Bases	Error (%)	Q20 (%)	Q30 (%)	GC Content (%)
AP45	D3	32139072	31426448	4.7G	0.03	97.47	93.16	64.07
	D4	28631614	28170950	4.2G	0.03	97.70	93.68	63.98
	D5	31557006	30794260	4.6G	0.03	97.72	93.81	64.05
	D6	30800544	30237518	4.5G	0.03	97.29	92.71	64.25
ASP1	D3	54352918	53216818	8G	0.03	96.45	91.23	65.51
	D5	57710476	56184754	8.4G	0.03	96.83	92.09	65.24
ASP2	D3	50036804	49042074	7.4G	0.03	97.46	92.84	48.30
	D5	43804448	42554860	6.4G	0.03	97.56	92.97	47.68
ASP4	D3	49352688	48519654	7.3G	0.03	97.34	93.03	50.24
	D5	51434492	50512410	7.6G	0.03	97.17	92.66	50.24

*Q20: percentages of bases with correct base recognition rates > 99% in total bases, Q30: percentages of bases with correct base recognition rates > 99.9% in total bases.*

**TABLE 2:** Statistics of assembled transcripts.

Strain name	Number of transcripts	Min. length (bp)	Average length (bp)	Max. length (bp)	Number of unigenes
AP45	23,881	201	1,288	22,247	23,871
ASP1	49,729	201	1,051	26,914	49,677
ASP2	19,940	201	1,888	19,081	19,932
ASP4	19,763	201	1,539	33,102	19,745

### Functional Classification of Unigenes

Gene Ontology (GO) is an international standardized gene functional classification system that defines genes according to three ontologies: molecular function, cellular component and biological process. For the four strains, 54.17% to 65.5% of unigenes were annotated in the GO database and assigned to 54 to 57 subcategories that shared similar frequency patterns among strains (Supplementary Figure 1). For all four strains, the majority of annotated unigenes were classified under the biological process (46.9%–47.89%), followed by cellular component (30.23%–31.44%), then molecular function (21.66%–21.97%). In the biological process category, the top two subcategories were ‘cellular process’ (20.93%–21.99%) and ‘metabolic process’ (18.45%–19.29%). In the cellular component category, the top two subcategories were ‘cell’ (18.04%–19.28%) and ‘cell part’ (18.04%–19.28%). In the molecular function category, the top two subcategories were ‘binding’ (46.21%–48.13%) and ‘catalytic activity’ (35.21%–36.86%).

Orthologous gene products were classified using COG and KOG. Between 27.52% and 34.22% of unigenes of the four strains were annotated and assigned to 25 KOG classifications (Supplementary Table 2). The frequency distribution of the classifications was very similar among the four strains (Supplementary Figure 2). For all four strains, the most unigenes were assigned to ‘General function prediction only’ (14.46% to 17.39%), followed by ‘Signal transduction mechanisms’ (12.54% to 15.04%), and ‘Posttranslational modification, protein turnover, chaperones’ (11.52% to 12.31%).

The characteristics of unigenes in metabolic pathways was estimated using KEGG. In total, 3,261 (AP45), 7,002 (ASP1), 2,917 (ASP2), and 2,994 (ASP4) unigenes were assigned to five categories: cellular processes (14.54% to 16.32%), environmental information processing (8.69% to 8.9%), genetic information processing (20.27 to 22.11), metabolism (33.21% to 36.62%) and organismal systems (19.58% to 21.02%). These five categories were further assigned to 32 KEGG pathways. For all four strains, the main metabolism terms were ‘Signal transduction’ (10.39% to 10.7%), ‘Translation’ (9.6% to 12.91%) and ‘Folding, sorting and degradation’ (8.27% to 10.42%). Other terms were also significant in individual strains, such as ‘Amino acid metabolism’ in ASP1 (7.95%), ‘Transport and catabolism’ in ASP2 (8.98%) and ‘Cell growth and death’ in ASP4 (9.05%).

The KEGG pathways related to the production of important potential industrial enzymes included ‘Amino acid metabolism,’ ‘Carbohydrate metabolism,’ ‘Lipid metabolism,’ ‘Metabolism of terpenoids and polyketides’ and ‘Xenobiotic biodegradation and metabolism’ (Table 3, Supplementary Figure 3). For all four strains, more unigenes were assigned to the first two pathways (4.98–5.99%), and the fewest to the last two pathways (0.72–1.21%).

**TABLE 3:** Number and proportion of unigenes classified into the five KEGG pathways, related to the production of potential industrial enzymes.

	*AP*45	*ASP*1	*ASP*2	*ASP*4
Amino acid metabolism	239(5.55%)	557(5.99%)	202(5.38%)	196(4.98%)
Carbohydrate metabolism	237(5.50%)	543(5.84%)	206(5.49%)	224(5.70%)
Lipid metabolism	139(3.23%)	284(3.06%)	114(3.04%)	115(2.92%)
Metabolism of terpenoids and polyketides	31(0.72%)	75(0.81%)	39(1.04%)	37(0.94%)
Xenobiotics biodegradation and metabolism	52(1.21%)	107(1.15%)	45(1.20%)	33(0.84%)

### Identification of Industrial Enzymes

Among the 17 targeted industrial enzymes, amylase and cellulase were absent in all four strains. For the remaining enzymes, homologous unigenes were found in at least one of the strains, and most of them were found in all four strains (Table 4).

**TABLE 4:** Number of unigenes of potential industrial enzymes identified in the four thraustochytrid strains.

Industrial enzymes	AP45	ASP1	ASP2	ASP4
Amylase	0	0	0	0
Alkaline phosphatase	1	3	3	6
Carboxylesterase	4	0	0	2
Cellulase	0	0	0	0
Chitinase	0	0	1	1
Dioxygenase	2	2	1	1
α-glucosidase	4	3	3	7
β-glucosidase	2	5	3	7
Ketosynthase	4	4	2	3
Laccase	1	1	1	1
L-asparaginase	1	1	1	1
Lipase	4	5	3	3
Monooxygenase	9	5	2	2
Peroxidase	2	3	3	2
Phytase	0	1	0	0
Protease	7	10	5	4
Urease	1	1	1	1

The enzymes with the 6 largest numbers of homologous unigenes found were glycohydrolase (α- and β-glucosidase), protease, monooxygenase, lipase, alkaline phosphatase and ketosynthase (34, 26, 18, 15, 13, 13, respectively), while phytase (only one in ASP1) and chitinase (one each in ASP2 and ASP4) were the two enzymes found with the fewest unigenes in the four strains. Laccase, L-asparaginase and urease had only one homologous unigene identified in each strain (Table 4).

**FIGURE 2:**
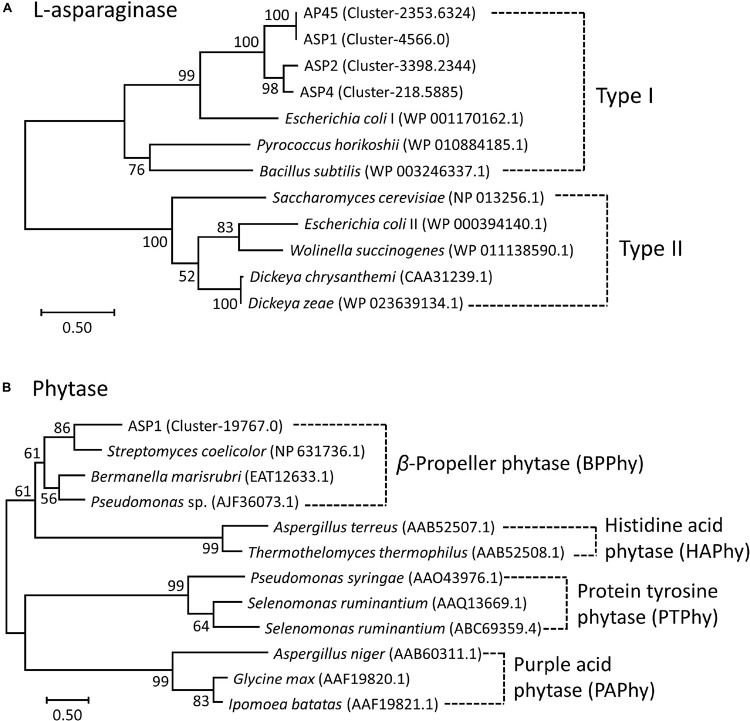
Maximum-Likelihood (ML) trees of **(A)** L-asparaginase and **(B)** Phytase. Node labels are bootstrap values (%) calculated with 5,000 replicate searches. Scale bars indicate the number of expected substitution per site with the LG + G + I and WAG + G distance model in **(A)** and **(B)**, respectively. Numbers within parentheses denote the NCBI Reference Sequence or GenBank ID.

LG + G + I and WAG + G models were selected as the best amino acid substitution models for L-asparaginase and phytase, respectively (Kumar et al., 2018). Based on the phylogenetic tree of L-asparaginase (Figure 2A), sequences of the four thraustochytrid strains were first grouped together, then formed a well-supported clade with bacteria *Escherichia coli* and *Bacillus subtilis*, and archaebacteria *Pyrococcus horikoshii*, which all belonged to Type I L-asparaginase. Complete sequence of phytase was only obtained in ASP1, with 479 amino acids. Based on the phylogenetic tree of phytase (Figure 2B), this sequence is sister to bacteria *Streptomyces coelicolor, Bermanella marisrubri*, and *Pseudomonas* sp., which all belong to the β-propeller phytase. Sequence alignment of amino acids of L-asparaginase and β-propeller phytase further confirmed the high conservation among the homologous genes (Supplementary Figures 4, 5).

### Detection of Enzymatic Activities

Table 5 shows the results of the enzyme assays. The activities of lipase, protease and alkaline phosphatase were detected in all four strains in both extra- and intra-cellular spaces. Urease activity was detected in the extracellular part only, while the L-asparaginase and α-glucosidase were observed only from crude cell lysates in all four thraustochytrids strains. The activities of amylase, cellulase, or phytase were not detected in any of the four strains.

**TABLE 5:** Detection of extracellular/lysate enzymatic activities in the four thraustochytrid strains.

Enzyme	AP45	ASP1	ASP2	ASP4
Urease	+ /−	+/−	+ /−	+/−
L-asparaginase	−/ +	−/ +	−/ +	−/ +
Phytase	−/−	−/−	−/−	−/−
Lipase	+ /+	+/+	+ /+	+/+
α-glucosidase	−/ +	−/ +	−/ +	−/ +
Alkaline phosphatase	+ /+	+/+	+ /+	+/+
Protease	+ /+	+/+	+ /+	+/+
Amylase	−/−	−/−	−/−	−/−
Cellulase	−/−	−/−	−/−	−/−

### Purification and Characterization of the Recombinant L-Asparaginase and Urease

**FIGURE 3:**
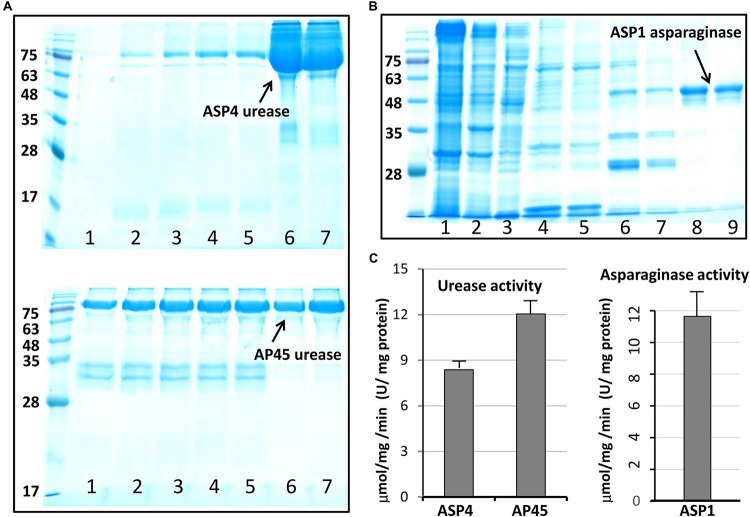
Purified urease and asparaginase and enzymatic activity analysis. **(A)** Soluble, high-level expression of Cr-ArsA2 in *E. coli* and purification by Ni-NTA chromatography. SDS-PAGE gel analysis: lane 1 to 5 – wash with 100 mM imidazole; lane 6 and 7 – elution with 250 mM imidazole. **(B)** SDS-PAGE gel analysis of purified L-asparaginase: lane 1 to 3, supernatant, pellet and flow through; lane 4 to 5 – wash with 50 mM imidazole; lane 6 and 7 – wash with 100 mM imidazole; lane 8 and 9 – elution with 250 mM imidazole. **(C)** Activity assay of purified urease and asparaginase. The experiment was performed by incubating about 2–5 μg enzyme in the reaction mixture (200 μl) containing 50 mM Tris-HCl at pH 7.0, 150 mM NaCl. The released product was measured colorimetrically, and quantified based on the standard curve. All reactions were done in triplicate.

This work selected four full-length ORFs, ASP1-L-asparaginase (Supplementary Figure 4), ASP1-phytase (Supplementary Figure 5), and AP45- and ASP4-urease (Supplementary Figure 6) for recombinant expression. Figure 3 shows the purification outcome from SDS-PAGE under reducing conditions. Milligram quantities of asparaginase and urease were successfully purified from one liter of *E. coli* culture. However, the ASP1 phytase could not be isolated, although the constructed plasmids were sequenced to determine the correct DNA sequence (Supplementary Figure 7). To test the enzymatic activity of asparaginase and urease, experiments were determined by using colorimetric assay. The purified L-asparaginase (∼12 μmol mg-1min-1, U/mg) and urease (∼10 μmol mg-1 min-1, U/mg) indicated clear activity of deamination of L-asparagine to aspartic acid and ammonia, and hydrolysis of urea into carbon dioxide and ammonia, respectively (Figure 3).

## Discussion

Seventeen potential industrial enzymes were surveyed, of which 15, excluding amylase and cellulase, homologous genes were found in at least one of the four strains (Table 4). The enzymes with the 6 largest numbers of homologous unigenes found were glycohydrolase (α- and β-glucosidase), protease, monooxygenase, lipase, alkaline phosphatase and ketosynthase, while phytase and chitinase were the two enzymes found in the fewest unigenes in the four strains. Laccase, L-asparaginase and urease had only one homologous unigene identified in each strain.

Various analyses were also performed for the measurement of enzymatic activities at day 5, and the results were mostly congruent with the transcriptome data. Most enzymatic activities, including urease, asparaginase, lipase, glucosidase, alkaline phosphatase and protease, were observed in all strains from extracellular or cell lysate, or both. This result is similar to that of a previous study in enzyme application, which concluded that thraustochytrids can produce polysaccharide hydrolyzing and degradative enzymes (Bremer and Talbot, 1995; Taoka et al., 2009). The four tested strains only exhibited L-asparaginase and α-glucosidase activities in cell lysate, revealing that these two enzymes are localized in the intracellular space. Previous studies suggested that *Thraustochytrium* and *Schizochytrium* strains could produce amylase or cellulase activity (Bremer and Talbot, 1995; Taoka et al., 2009). However, experimental results did not show the specific polysaccharides-hydrolyzing functions (Table 5), suggesting that the four isolated thraustochytrids could not produce amylase or cellulase. This observation is supported by the transcriptome data, which showed no homologous genes of these two enzymes in any of the four strains. Finally, phytase activity was not detected in the extracellular space, although its homolog, possessing a signal peptide (27 amino acid-long), was determined in ASP1 transcriptome. This result is concordant with the very low transcript copy (Fragments Per Kilobase of transcript per Million mapped reads = 5.27 at day 5) of the phytase gene. Phosphate starvation was suggested to induce phytase formation in most microbes (Reale et al., 2007), suggesting that a modified culture medium might improve the phytase expression in thraustochytrids.

Urease is a nickel dependent ammonium permerase that catalyzes the hydrolysis of urea into carbon dioxide (CO_2_) and ammonia (NH_3_). Urease is widely distributed in soils, feces and manure (mixture of urine and feces), and is generally found in bacteria, fungi, and plants. Most bacterial ureases are heteropolymers that have a native molecular weight of ∼200 to 600 kDa, while ureases in plants and fungi are composed of homooligomers of ∼90 kDa identical subunits. The four thraustochytrid ureases retrieved in this study likely share the structural characteristics with plants and fungi based on similar molecular weight and high sequence conservation (Supplementary Figure 6). Although the bacterial and plant (or fungal) ureases have similar catalytic mechanism and certain conserved sequence features, bacterial ureases generally possess much higher activities (∼1,000 to 5,500 U/mg) than that in plants (<50 U/mg) (Mobley et al., 1995). One of the most frequently studied bacterial urease is the *Helicobacter pylori*, a Gram-negative spiral bacterium, which has been implicated in gastrointestinal diseases, including gastritis, peptic ulcers, stomach cancer, etc. This bacterium generally produces a large quantity of urease (∼15% of total proteins) with high activity for urea hydrolysis (∼1,000 to 3,000 U/mg), which is important for its survival and pathogenesis within the gastric lumen after neutralizing gastric acid with ammonia (Ha et al., 2001). Urease activity is considered a medical evaluation and diagnostic in the virulence and pathogenesis, including urinary stones, gastritis, peptic ulceration and hepatic encephalopathy (Griffith, 1979; Mobley, 1996). Urease activity has also been shown to have potent insecticidal activity against cowpea weevil *Callosobruchus maculatus* and the green stinkbug *Nezara viridula*, and antifungal activities against *Curvularia lunata*, *Fusarium solani*, *Fusarium oxysporum*, etc. (Follmer et al., 2004; Becker-Ritt et al., 2007; Balasubramanian et al., 2013). This study identified one urease enzyme in each thraustochytrid strain, and harvested milligram quantities of active AP45- and ASP4-ureases from one liter of *E. coli* culture (Figure 3). The purified recombinant ureases exhibited clear activity of hydrolysis of urea into carbon dioxide and ammonia (∼10 U/mg). This observation is comparable to those reported in the commercial urease from jack bean with activity ∼1–50 U/mg based on different production batches (Sigma-Aldrich, catalog no. 94280, 94281 and U1500). Higher activity of thraustochytrid ureases under optimal reaction conditions is plausible because these microbes probably grow on ammonia, the decomposed product of urea, as a nitrogen source in mangroves (Holguin et al., 2001; Taoka et al., 2009).

For the first time, this study reports the presence in thraustochytrids of two well-recognized industrial enzymes, L-asparaginase and phytase, which have been previously reported from a wide spectrum of taxonomic groups, ranging from microbes and plants to animals. Asparaginase is an enzyme responsible for the metabolism of L-asparagine, by catalyzing the hydrolysis of L-asparagine into L-aspartic acid and ammonia in living organisms. L-asparaginase has been applied widely in medical applications, the treatment of diseases (e.g., acute lymphoblastic leukemia, non-Hodgkin’s lymphoma and acute myeloid leukemia), and the reduction of carcinogenic acrylamide in food industries (Boyse et al., 1967; Narta et al., 2007; Pedreschi et al., 2008; Costa et al., 2016). Although several types of L-asparaginase have been identified from their amino acid sequences and biochemical properties, only two types (Type I and II) have been found in microbes (Cedar and Schwartz, 1967; Borek and Jaskólski, 2001; Izadpanah Qeshmi et al., 2018). Type I functions as a dimer cytoplasmic enzyme, while Type II prefers to form a tetramer located at periplasm (Cedar and Schwartz, 1967; Jerlström et al., 1989; Borek and Jaskólski, 2001). Although Type II L-asparaginase has more medical applications, Type I isolated from archaebacteria *Thermococcus kodakarensis* is reported to be thermostable and highly active at the high temperature of 85°C (Chohan and Rashid, 2013; Guo et al., 2017). This study obtained complete sequences of L-asparaginase from all four strains. The sequences were about 371–382 amino acids in length, and all belonged to the Type I family (Figure 2 and Supplementary Figure 4). The purified ASP1 L-asparaginase exhibited a similar activity of deamination (∼12 U/mg) to *Bacillus coagulans* (∼11 U/mg) (Kumar and Sobba, 2013), but lower activity compared with other microbes such as commercial asparaginase from *E. coli* (∼100 U/mg) (Sigma-Aldrich, catalog no. A3809), *Staphylococcus* sp. OJ82 (∼100 U/mg), a Gram-positive bacteria isolated from seafood (Han et al., 2014), or *Thermococcus kodakaraensis*, a species of thermophilic archaea isolated from the Solfatara, a shallow volcano crater, exhibiting highest ever reported enzyme activity (∼2,350 U/mg) (Chohan and Rashid, 2013). Like the Type I homologue found in *T. kodakarensis*, thraustochytrid L-asparaginases could be tolerant to extreme conditions for inhabiting at mangroves where temperature, salinity, and pH fluctuate constantly (Chohan and Rashid, 2013). For future works, besides searching for optimal reaction conditions, such as pH, temperature, salt stress and metal ions effect, in order to obtain the mutants with enhanced catalytic efficiency, the enzyme engineering by directed evolution for generating random mutations of the four isolated L-asparaginases should be further studied.

Phytase can delay waste disposal with high phosphate by hydrolyzing phosphomonoester bonds from phytic acid, which is the major form of phosphorus in many cereals, grain legumes and oil seed crops (Irving and Cosgrove, 1974). Bacterial phytase is found in the intestines of ruminant animals, which makes the hosts able to digest phytic acid in grains as a source of phosphorus. Therefore, phytases have been widely applied in animal feed industries and bioremediation. Phytases play different physiological roles in living organisms. Plant phytases are responsible for the degradation of phytic acid during germination for plant growth and development, while phytase formation in most microbes is induced in response to phosphate starvation (Reale et al., 2007). Four types of phytases, namely Histidine Acid Phosphatases (HAPhy), β-Propeller Phytases (BPPhy), Protein Tyrosine Phosphatases (PTPhy) and Purple Acid Phytases (PAPhy), have been identified from chemical structure and catalytic mechanism (Konietzny and Greiner, 2002; Mullaney and Ullah, 2003; Chu et al., 2004; Lei et al., 2013; Irshad et al., 2017). As HAPhy, BPPhy and PAPhy are acid phosphatases, they work effectively in the digestive tract of monogastric animals (Lei and Stahl, 2001; Chiera et al., 2004). HAPhy are the best known and marketed acid phosphatases; BPPhy are calcium-dependent and can function under neutral and alkaline conditions (Cheng and Lim, 2006; Kumar et al., 2017); PTPhy are mostly reported from anaerobic ruminal bacteria (Yanke et al., 1999); PAPhy are metalloenzymes (Schenk et al., 2000). The complete sequence of ASP1 phytase belongs to the BPPhy family (Figure 2), and is about 479 amino acids long with at least 20 conserved residues within the active sites for dephosphorylation and calcium binding (Supplementary Figure 5A). Its structural modeling clearly shows a canonical folding architecture of a propeller with six blades (Supplementary Figure 5B). Supplementary Figure 7 shows the SDS PAGE and western blot analysis of the expression of ASP1 phytase. However, the purification of ASP1 phytase was not successful, likely due to the expression difficulties of the 27 amino acid-long N-terminal signal peptide and an unusual hydrophobic transmembrane helix predicted at the very C-terminus (approximately between amino acid 427 and 446) (Supplementary Figures 5, 7). Further experiments, including PCR determination of correct full-length ORF for translation, combined with biochemical and structural analyses will be required to identify the exact role of phytase in thraustochytrids.

Previous studies have suggested that thraustochytrids could play an important role in enhancing carbon and nitrogen cycling through organic degradation in the mangroves and a carbon conveyer between land and sea (Bremer and Talbot, 1995; Kimura et al., 1999; Holguin et al., 2001; Taoka et al., 2009; Kagami et al., 2014; Dellero et al., 2018b). Indeed, our data indicate that thraustochytrids from the coastal water of Taiwan could produce abundant and diverse enzymes. Like other microbes, thraustochytrids can grow easily and inexpensively, and can produce sufficient quantity of enzymes. Therefore, the diverse marine ecosystem of Taiwan is a source of marketable high-performance enzymes with industrial applications. Observation results highlight the diverse enzymes for wide-range applications in thraustochytrids. This study provides broad implications for food and traditional industries, as well as in implementation of sustainable use of biodiversity-based resources, and thus could provide a cleaner and healthier environment for all life on earth.

## Data Availability Statement

The datasets presented in this study can be found in online repositories. The names of the repository/repositories and accession number(s) can be found in the article/ Supplementary Material.

## Author Contributions

H-CL and H-YC conceived the project. W-HL carried out the transcriptome experiments and analyses. C-CC, T-HC, and Y-HL carried out the protein experiments and analyses. M-DH, W-MC, and J-SC helped supervise the project. H-CL took the lead in writing the manuscript. All authors provided critical feedback that helped shape the research, analyses, and manuscript.

## Conflict of Interest

The authors declare that the research was conducted in the absence of any commercial or financial relationships that could be construed as a potential conflict of interest.

## Footnotes

1 https://www.geneious.com
