# Supplementary material for: Diverse Enzymes With Industrial Applications in Four Thraustochytrid Genera
Source: Front Microbiol. 2020 Oct 20;11:573907. doi: 10.3389/fmicb.2020.573907 (PMC7641610; doi:10.3389/fmicb.2020.573907)
Supplement: Supplementary file 1 [file Data_Sheet_1.PDF]

Supplementary Material

Table 1. Amino acid sequences of industrial enzymes applied as queries for searching homologous genes in transcriptome datasets of the four thraustochytrid strains.

Industrial enzymes (EC Number)	GenBank or PDB ID	Description	Species
Alkaline phosphatase (EC: 3.1.3.1)	OJJ16279.1	Alkaline phosphatase	marine bacterium AO1-C
	XP_009308464.1	Alkaline phosphatase	*Trypanosoma grayi*
Amylase (EC: 3.2.1.-)	AAA97431.1	Amylase	*Streptococcus equinus*
	AAX85453.1	Amylase	*Bacillus* sp. WPD616
	AGD88873.1	Amylase	Flavobacteriaceae bacterium BPA
	BAT46646.1	Amylase	*Paenibacillus* sp. SSG-1
	CAD20312.1	Amylase	*Tetraodon nigroviridis*
Carboxylesterase (EC:3.1.1.1)	5MIF_A	Chain A, 'carboxyl Esterase 2	*Tuber melanosporum* Mel28
	5MII_A	Chain A, Carboxyl esterase 2	*Tuber melanosporum* Mel28
	CAI79615.1	Carboxylesterases	*Cryptococcus neoformans* var. neoformans
	EED77770.1	Carboxylesterase-like protein, partial	*Postia placenta* Mad-698-R
	KMQ48452.1	Carboxylesterase type B, active site	*Trichophyton rubrum*
Cellulase (EC: 3.2.1.4)	ACH91035.1	Cellulase	*Talaromyces funiculosus*
	OGM46452.1	Cellulase	*Aspergillus bombycis*
	XP_018656481.1	Cellulase	*Trichoderma gamsii*
Chitinase (EC:3.2.1.14)	KOO33516.1	Chitinase	*Chrysochromulina sp.* CCMP291
	XP_007508704.1	Chitinase	*Bathycoccus prasinos*
	XP_015656325.1	Chitinase	*Leptomonas pyrrhocoris*
Dioxygenase (EC:1.13.11.-.)	EER43885.1	Dioxygenase	*Histoplasma capsulatum* H143
	EKD12237.1	Dioxygenase	*Marssonina brunnea* f. sp. 'multigermtubi' MB_m1
	GAT23367.1	Dioxygenase	*Aspergillus luchuensis*
	GAT23610.1	Dioxygenase	*Aspergillus luchuensis*
α -glycoside hydrolase (EC: 3.2.1.-)	GBG30184.1	Neutral alpha-glucosidase AB	*Hondaea fermentalgiana*
	WP_125559381.1	Alpha-glucosidase	*Pseudoalteromonas rubra*
β -glycoside hydrolase (EC: 3.2.1.-)	GBG34762.1	Beta-glucosidase cel3A	*Hondaea fermentalgiana*
	OQR94490.1	Glycoside hydrolase	*Achlya hypogyna*
Ketosynthase	AAX39746.1	type I polyketide synthase-like protein AS1-1L, partial	*Karenia brevis*
	AAX39747.1	type I polyketide synthase-like protein AT2-10L, partial	*Karenia brevis*
	AAX39748.1	type I polyketide synthase-like protein AT2-15, partial	*Karenia brevis*
	AAX86994.1	type I polyketide synthase-like, partial	bacterium
	AAX86995.1	type I polyketide synthase-like, partial	bacterium
	AAX86996.1	type I polyketide synthase-like, partial	bacterium
	AAX86997.1	type I polyketide synthase-like, partial	bacterium
Laccase (EC:1.10.3.2)	GBG34229.1	Laccase-1	*Hondaea fermentalgiana*
L-asparaginase (EC:3.5.1.1)	EMS12433.1	L-asparaginase	*Entamoeba histolytica* HM-3:IMSS

Lipase (EC: 3.1.1.-)	KEG08385.1	Lipase	*Trypanosoma grayi*
	OWZ21788.1	Lipase	*Phytophthora megakarya*
Monooxygenase (EC:1.13.12.-.)	AUB27736.1	Monooxygenase	*Cryptococcus neoformans* var. grubii
	RMJ27688.1	Monooxygenase	*Phialosimplex sp.* HF37
	RMZ48437.1	Monooxygenase	*Aspergillus flavus*
	RNJ52195.1	Monooxygenase	*Verticillium nonalfalfae*
	XP_026622935.1	Monooxygenase	*Aspergillus welwitschiae*
Peroxidase (EC:1.11.1.-)	AAN65867.1	Peroxidase	*Pseudomonas putida* KT2440
	ALJ81860.1	Peroxidase	*Ketogulonicigenium vulgare*
	ATX76034.1	Peroxidase	*Reinekea forsetii*
	OQZ85631.1	Peroxidase	*Thioclava electrotropha*
Phytase (EC 3.1.3.-)	WP_015206240.1	Phytase	*Cylindrospermum stagnale*
Protease (EC:3.4.21.-)	EKU20638.1	Protease	*Nannochloropsis gaditana* CCMP526
	ELR24393.1	Protease	*Acanthamoeba castellanii* str. Neff
	EWM25835.1	Protease	*Nannochloropsis gaditana*
	XP_004354538.1	Protease	*Acanthamoeba castellanii* str. Neff
	XP_009521710.1	Protease	*Phytophthora sojae*
Urease (EC: 3.5.1.5)	KAE9345202.1	Urease	*Phytophthora fragariae*
	OWZ22462.1	Urease	*Phytophthora megakarya*
	XP_005786442.1	Urease	*Emiliania huxleyi* CCMP1516

Table 2. Number and percentage of unigenes successfully annotated in the seven public databases. (A) AP45, (B) ASP1, (C) ASP2, (D) ASP4.

(A)

	Number of Unigenes	Percentage (%)
Annotated in NR	8636	36.17
Annotated in NT	2300	9.63
Annotated in KO	3261	13.66
Annotated in SwissProt	9019	37.78
Annotated in PFAM	13162	55.13
Annotated in GO	13187	55.24
Annotated in KOG	6699	28.06
Annotated in all Databases	1323	5.54
Annotated in at least one Database	14751	61.79
Total Unigenes	23871	100

(B)

	Number of Unigenes	Percentage (%)
Annotated in NR	17105	34.43
Annotated in NT	4135	8.32
Annotated in KO	7002	14.09
Annotated in SwissProt	18697	37.63
Annotated in PFAM	26838	54.02
Annotated in GO	26912	54.17
Annotated in KOG	13673	27.52
Annotated in all Databases	2277	4.58
Annotated in at least one Database	30952	62.3
Total Unigenes	49677	100

(C)

	Number of Unigenes	Percentage (%)
Annotated in NR	8446	42.37
Annotated in NT	669	3.35
Annotated in KO	2917	14.63
Annotated in SwissProt	8851	44.4
Annotated in PFAM	11625	58.32
Annotated in GO	11634	58.36
Annotated in KOG	6529	32.75
Annotated in all Databases	487	2.44
Annotated in at least one Database	12900	64.72
Total Unigenes	19932	100

(D)

	Number of Unigenes	Percentage (%)
Annotated in NR	8899	45.06
Annotated in NT	1014	5.13
Annotated in KO	2994	15.16
Annotated in SwissProt	9307	47.13
Annotated in PFAM	12916	65.41
Annotated in GO	12933	65.5
Annotated in KOG	6757	34.22
Annotated in all Databases	523	2.64
Annotated in at least one Database	14517	73.52
Total Unigenes	19745	100

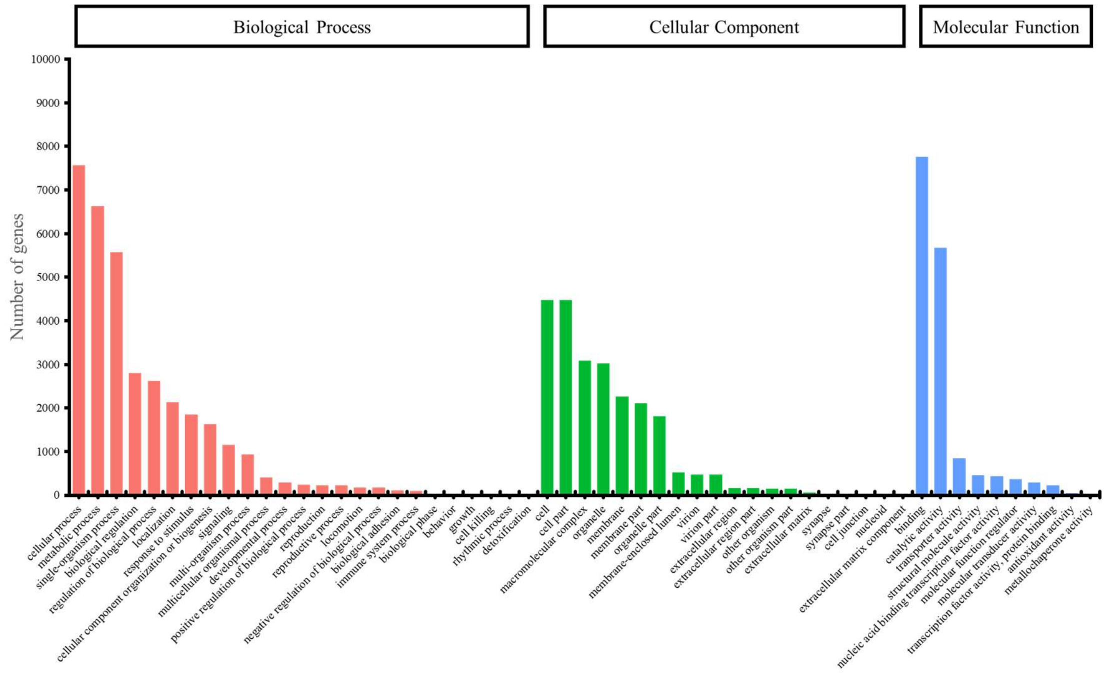
Biological Process
Cellular Component
Molecular Function
Number of genes

(A)

(B)

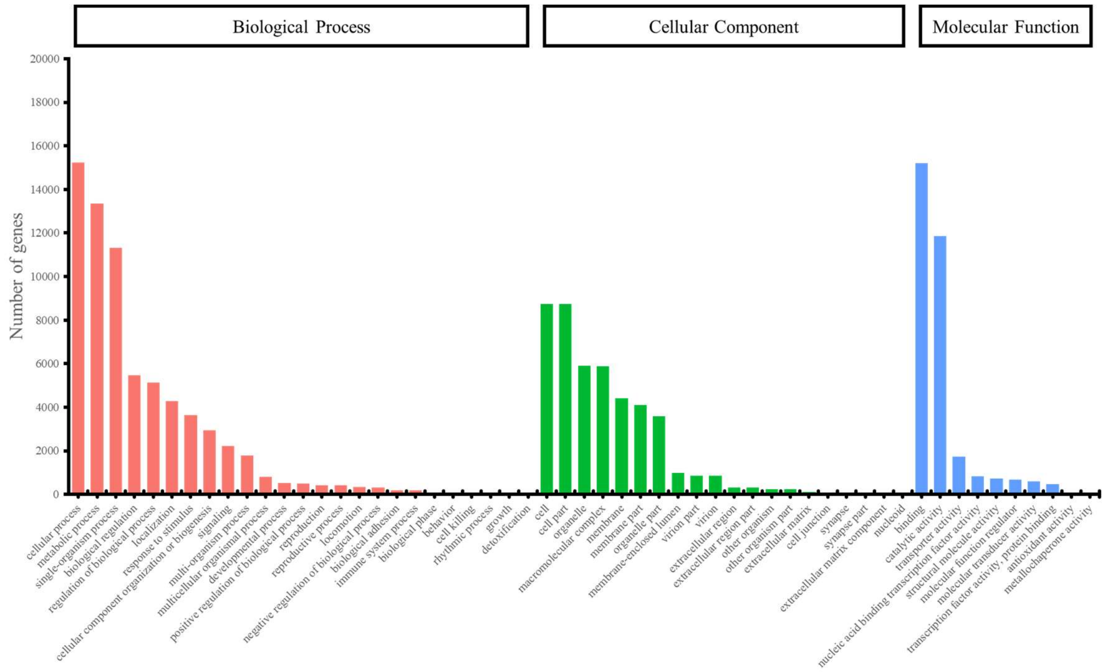
Biological Process
Cellular Component
Molecular Function
Number of genes

(C)

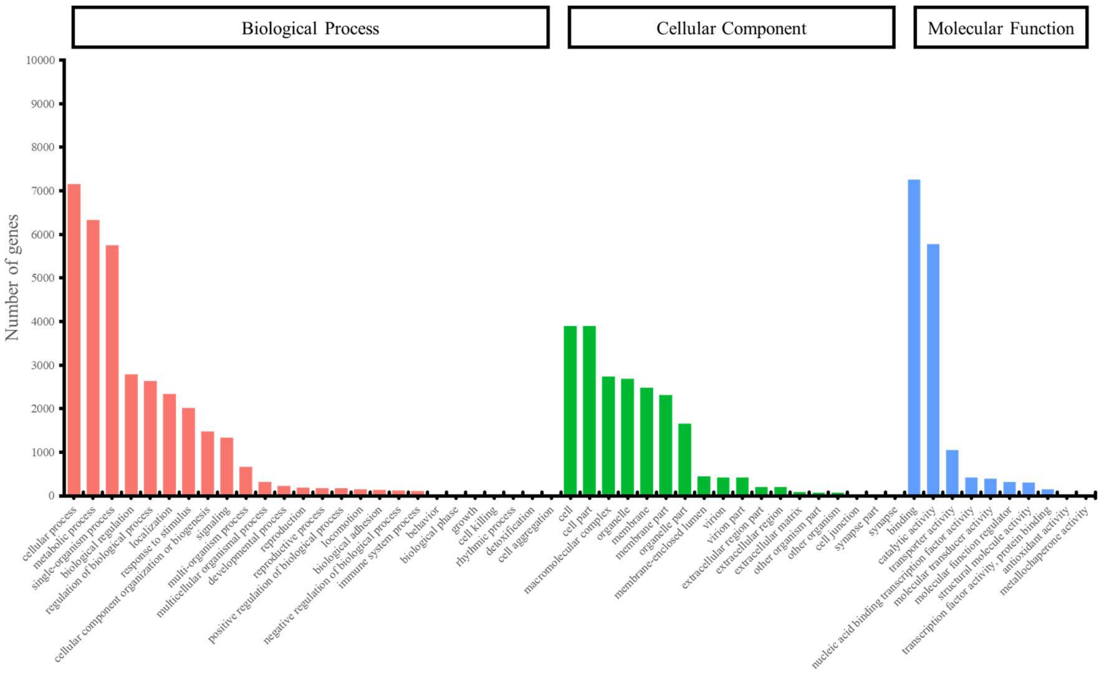

(D)

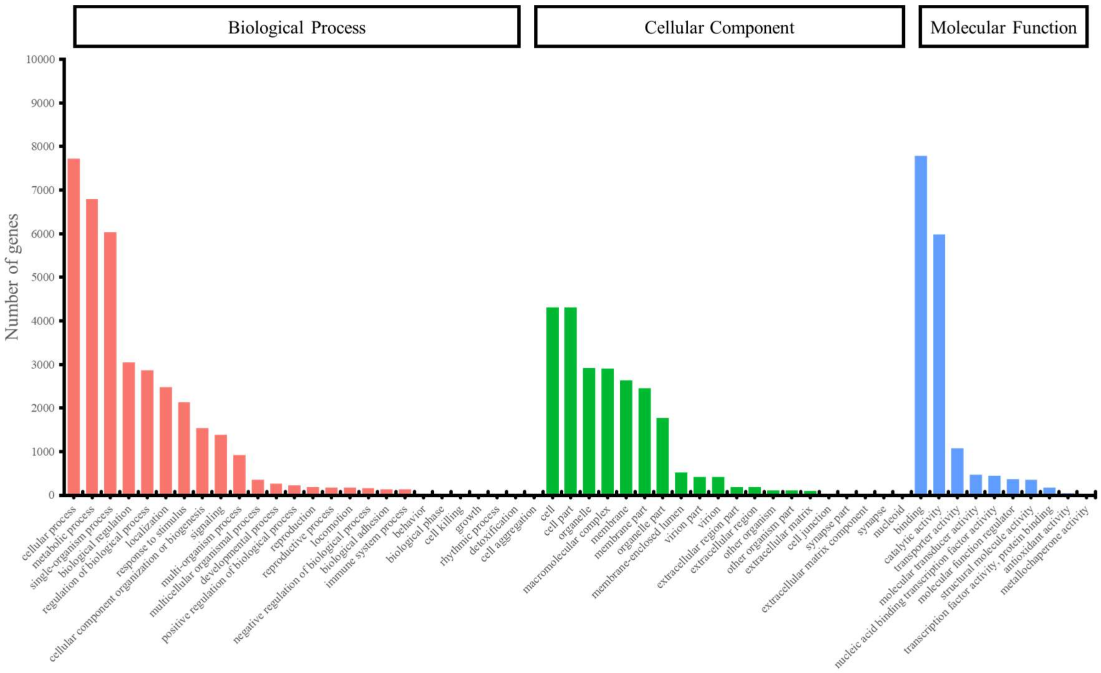

Fig. 1. GO classifications of assembled unigenes. (A) AP45, (B) ASP1, (C) ASP2, (D) ASP4.

(A)

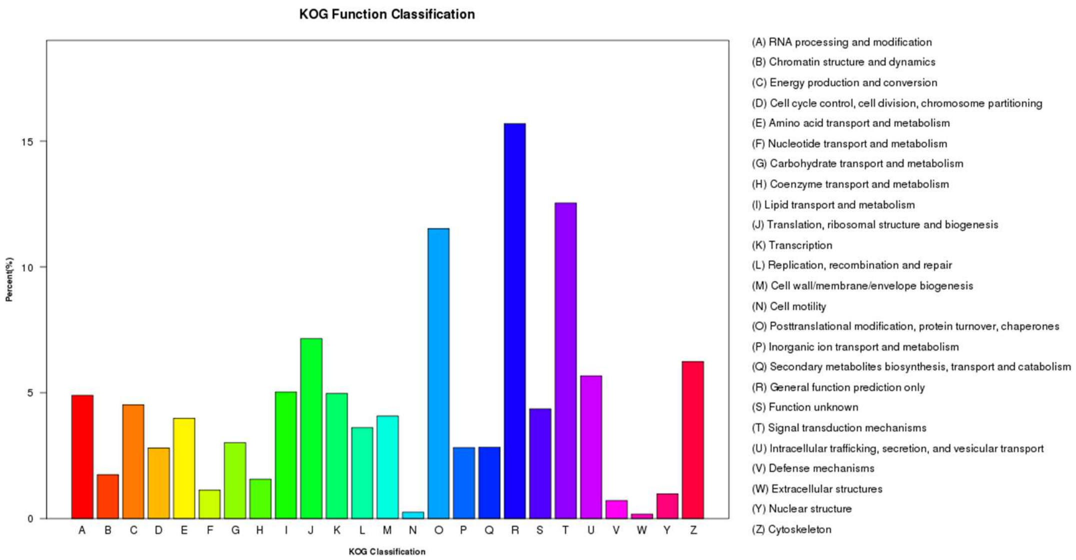
KOG Function Classification
Percent(%)
0
5
10
15
A B C D E F G H I J K L M N O P Q R S T U V W Y Z
KOG Classification
(A) RNA processing and modification
(B) Chromatin structure and dynamics
(C) Energy production and conversion
(D) Cell cycle control, cell division, chromosome partitioning
(E) Amino acid transport and metabolism
(F) Nucleotide transport and metabolism
(G) Carbohydrate transport and metabolism
(H) Coenzyme transport and metabolism
(I) Lipid transport and metabolism
(J) Translation, ribosomal structure and biogenesis
(K) Transcription
(L) Replication, recombination and repair
(M) Cell wall/membrane/envelope biogenesis
(N) Cell motility
(O) Posttranslational modification, protein turnover, chaperones
(P) Inorganic ion transport and metabolism
(Q) Secondary metabolites biosynthesis, transport and catabolism
(R) General function prediction only
(S) Function unknown
(T) Signal transduction mechanisms
(U) Intracellular trafficking, secretion, and vesicular transport
(V) Defense mechanisms
(W) Extracellular structures
(Y) Nuclear structure
(Z) Cytoskeleton

(B)

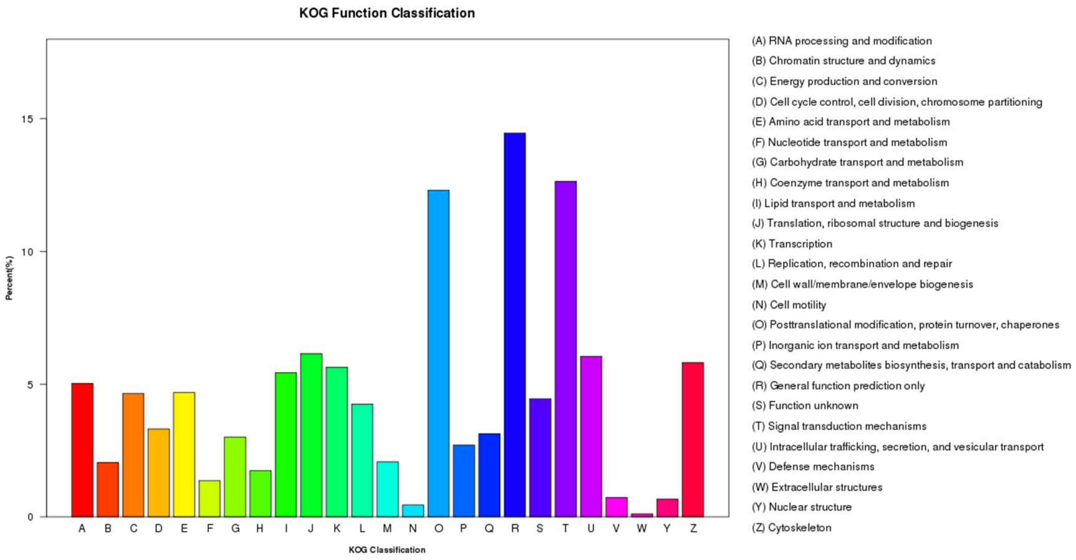
KOG Function Classification
Percent(%)
0
5
10
15
A B C D E F G H I J K L M N O P Q R S T U V W Y Z
KOG Classification
(A) RNA processing and modification
(B) Chromatin structure and dynamics
(C) Energy production and conversion
(D) Cell cycle control, cell division, chromosome partitioning
(E) Amino acid transport and metabolism
(F) Nucleotide transport and metabolism
(G) Carbohydrate transport and metabolism
(H) Coenzyme transport and metabolism
(I) Lipid transport and metabolism
(J) Translation, ribosomal structure and biogenesis
(K) Transcription
(L) Replication, recombination and repair
(M) Cell wall/membrane/envelope biogenesis
(N) Cell motility
(O) Posttranslational modification, protein turnover, chaperones
(P) Inorganic ion transport and metabolism
(Q) Secondary metabolites biosynthesis, transport and catabolism
(R) General function prediction only
(S) Function unknown
(T) Signal transduction mechanisms
(U) Intracellular trafficking, secretion, and vesicular transport
(V) Defense mechanisms
(W) Extracellular structures
(Y) Nuclear structure
(Z) Cytoskeleton

(C)

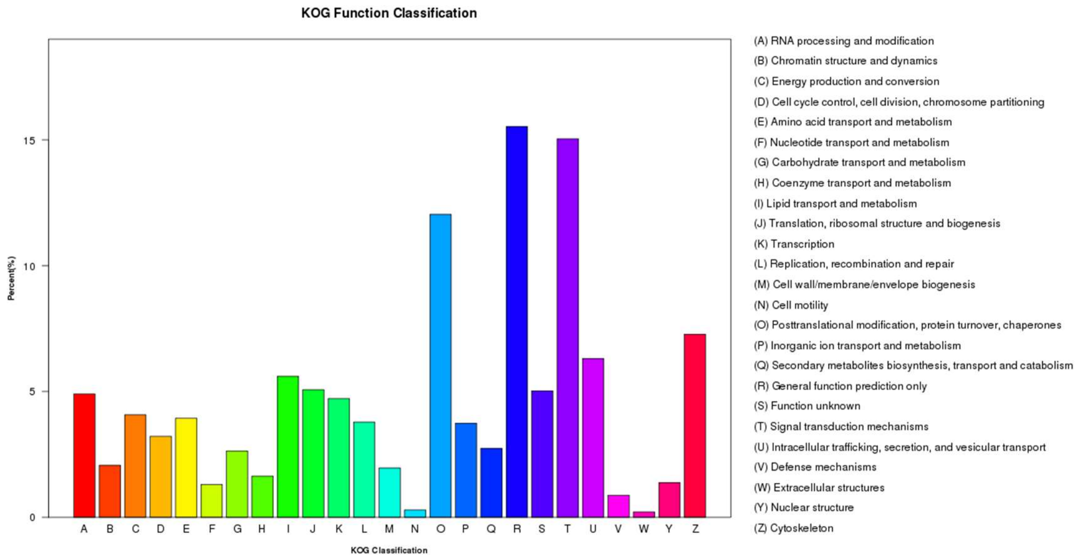

(D)

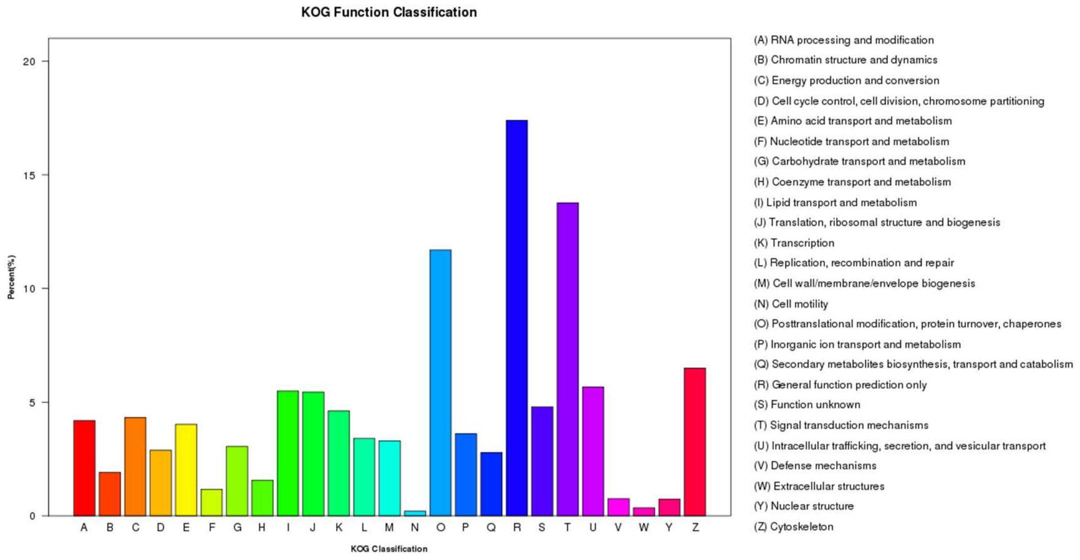

Fig. 2. KOG/COG classification of unigenes. (A) AP45, (B) ASP1, (C) ASP2, (D) ASP4.

(A)

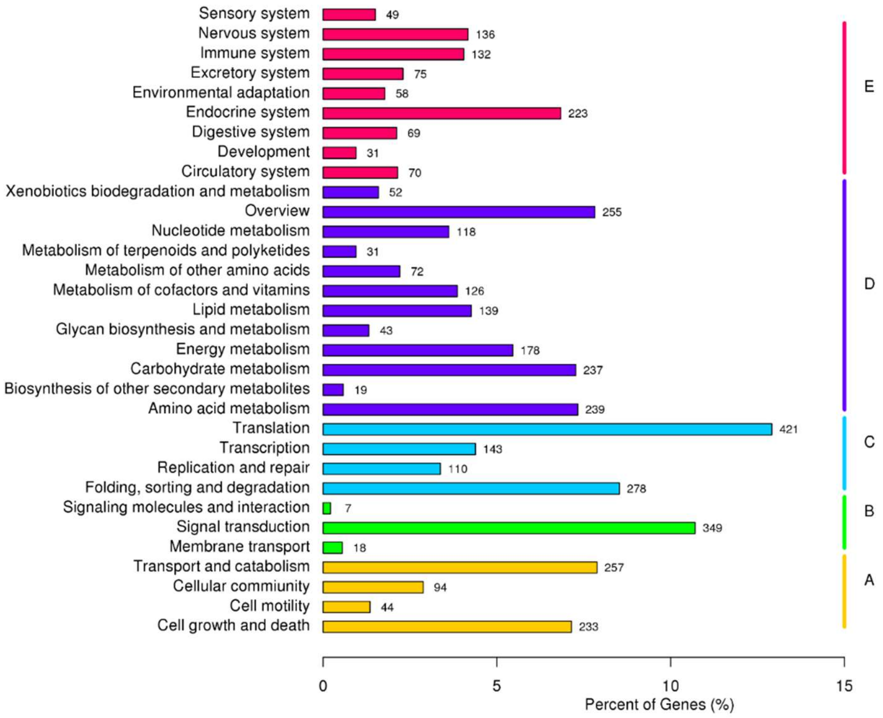
Sensory system 49
Nervous system 136
Immune system 132
Excretory system 75
Environmental adaptation 58
Endocrine system 223
Digestive system 69
Development 31
Circulatory system 70
Xenobiotics biodegradation and metabolism 52
Overview 255
Nucleotide metabolism 118
Metabolism of terpenoids and polyketides 31
Metabolism of other amino acids 72
Metabolism of cofactors and vitamins 126
Lipid metabolism 139
Glycan biosynthesis and metabolism 43
Energy metabolism 178
Carbohydrate metabolism 237
Biosynthesis of other secondary metabolites 19
Amino acid metabolism 239
Translation 421
Transcription 143
Replication and repair 110
Folding, sorting and degradation 278
Signaling molecules and interaction 7
Signal transduction 349
Membrane transport 18
Transport and catabolism 257
Cellular commiunity 94
Cell motility 44
Cell growth and death 233
E
D
C
B
A
0
5
10
15
Percent of Genes (%)

(B)

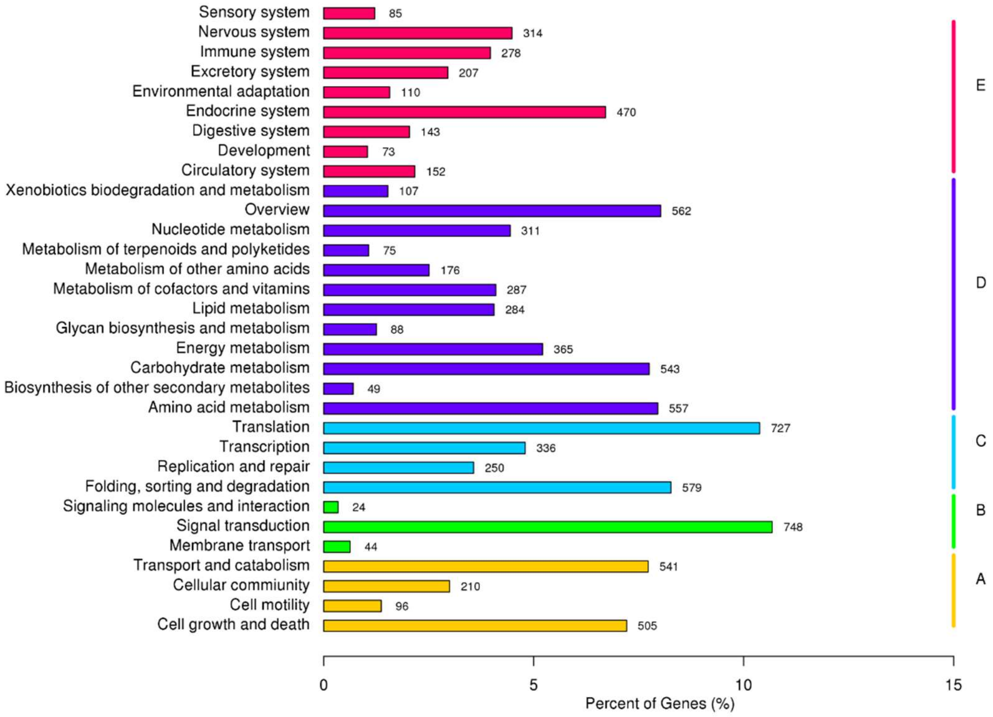
Sensory system 85
Nervous system 314
Immune system 278
Excretory system 207
Environmental adaptation 110
Endocrine system 470
Digestive system 143
Development 73
Circulatory system 152
Xenobiotics biodegradation and metabolism 107
Overview 562
Nucleotide metabolism 311
Metabolism of terpenoids and polyketides 75
Metabolism of other amino acids 176
Metabolism of cofactors and vitamins 287
Lipid metabolism 284
Glycan biosynthesis and metabolism 88
Energy metabolism 365
Carbohydrate metabolism 543
Biosynthesis of other secondary metabolites 49
Amino acid metabolism 557
Translation 727
Transcription 336
Replication and repair 250
Folding, sorting and degradation 579
Signaling molecules and interaction 24
Signal transduction 748
Membrane transport 44
Transport and catabolism 541
Cellular commiunity 210
Cell motility 96
Cell growth and death 505
E
D
C
B
A
0
5
10
15
Percent of Genes (%)

(C)

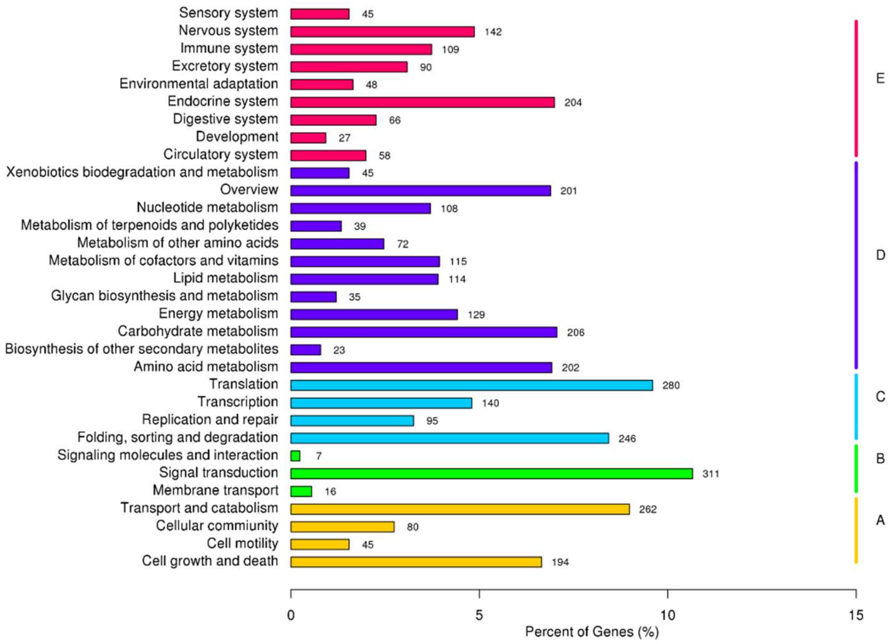

(D)

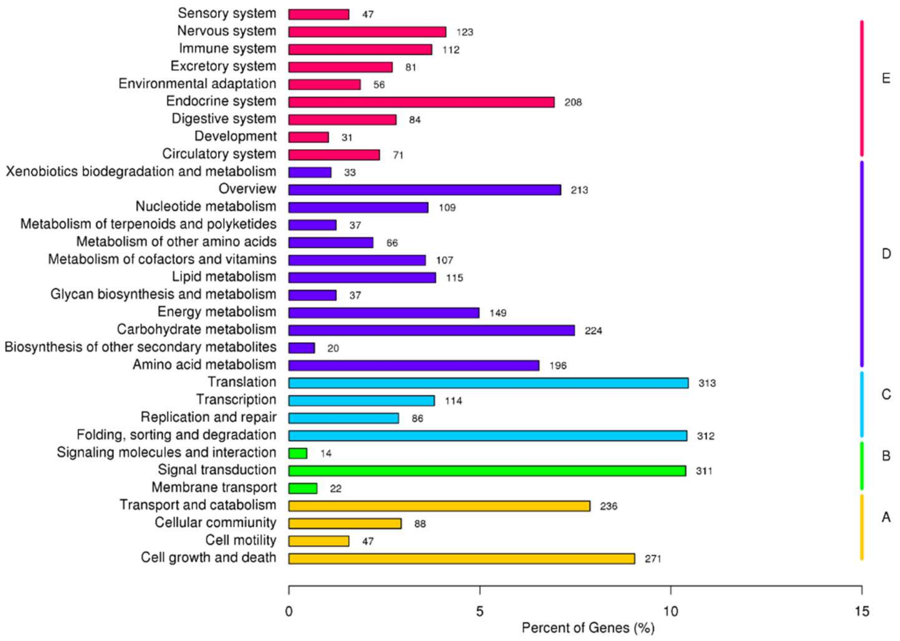

Fig. 3. KEGG classification of unigenes. (A) AP45, (B) ASP1, (C) ASP2, (D) ASP4. The capital letters against the colored bars indicate five main categories: A: cellular processes, B: environmental information processing, C: genetic information processing, D: metabolism, and E: organism systems.

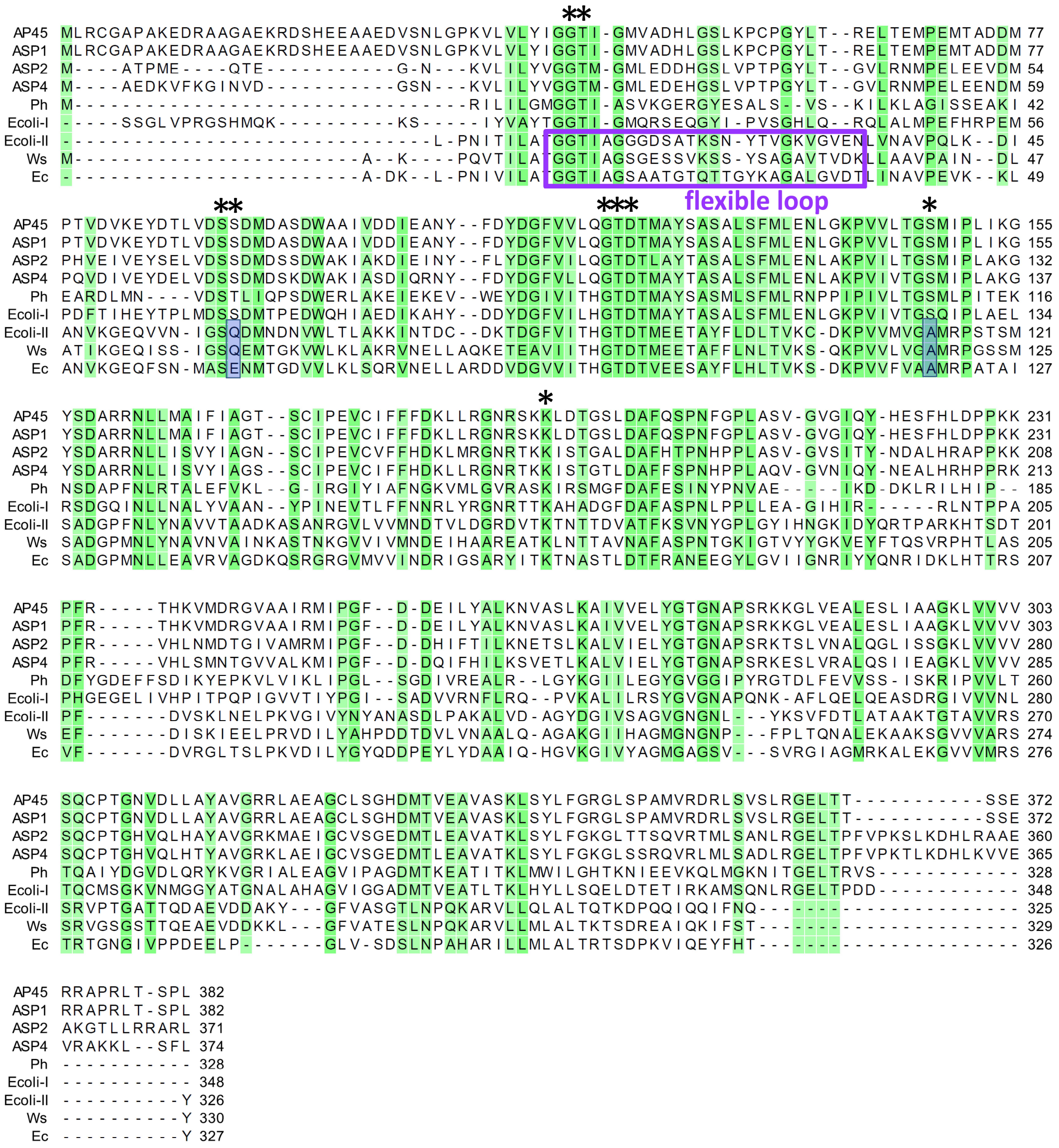

Fig. 4. Complete alignment of amino acid of Type I and II L-asparaginase enzymes. L-asparaginase enzymes could be divided into type I and II based on the flexible loop (blue rectangle) and certain conserved active site residues (blue boxes) (Yao et al., 2005). Type I enzymes include AP45, ASP1, ASP2, ASP4, *Pyrococcus horikoshii* (PDB ID: 1WLS) (Yao et al., 2005) and *Escherichia coli* I (PDB ID: 6NXD) (Lubkowski et al., 2019). On the other hand, *Escherichia coli* II (PDB ID: 3ECA) (Swain et al., 1993), *Wolinella succinogenes* (PDB ID: 1WSA) (Lubkowski et al., 1996) and *Erwinia chrysanthemi* (PDB ID: 1O7J) (Lubkowski et al., 2003) are belonged to Type II enzyme. The conserved amino acids are colored in green. The residues involved in catalysis are indicated by asterisks.

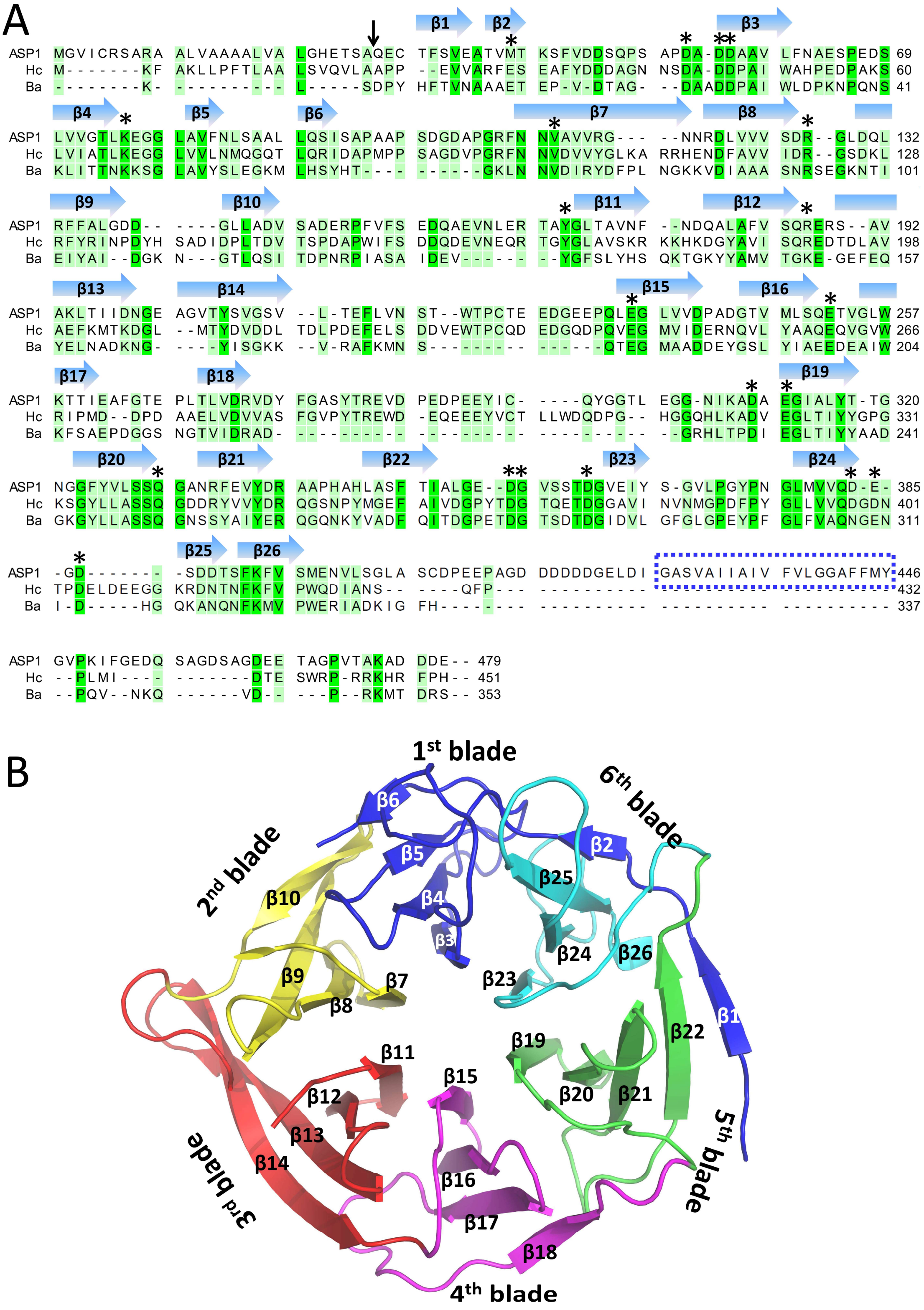

Fig. 5. Structural modelling of ASP1 phytase and alignment of the amino acid sequences of the phytase enzymes. (A) Multiple sequence alignment of phytase enzymes. ASP1 phytase is aligned

with the *Hahella chejuensis* (phytase: WP_011397470.1) and *Bacillus amyloliquefaciens* (PDB ID: 1CVM) (Ha et al., 2000). The conserved amino acids are colored in green. The upper 26 blue arrows indicate the β-strand regions based on the ASP1 phytase model and *B. amyloliquefaciens* phytase crystal structure (PDB ID: 1CVM) (Ha et al., 2000). Twenty conserved residues within the catalytic site for dephosphorylation and calcium binding are indicated by asterisks. Black arrow indicates the prediction of signal peptide cleavage site. The prediction of an unusual hydrophobic transmembrane helix at very C-terminus is highlighted by a blue dashed rectangle. (B) The ribbon diagram of the structural model for ASP1 phytase. Homology modeling was performed based on the known 3D structure of phytase from *B. amyloliquefaciens* (PDB ID: 1CVM). The predicted model shows a canonical folding architecture of a propeller with six blades. Twenty six β-strands, labeled from 1 to 26, are shown as strand cartoon and in different colors.

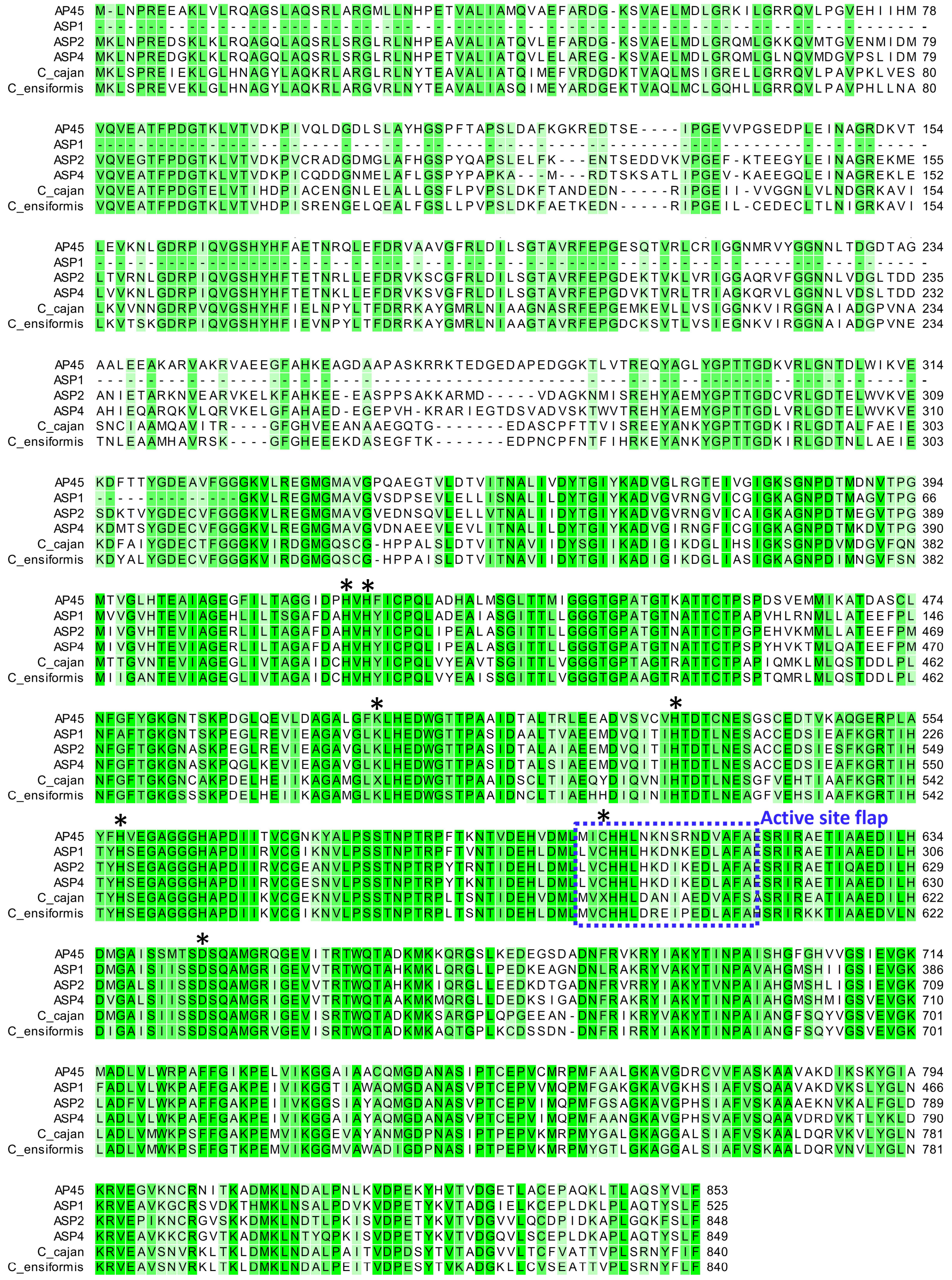

Fig. 6. Multiple sequence alignment of urease enzymes. The amino acid sequence alignment of five urease enzymes, includes four isolated Thraustochytrids strains, pigeonpea (*Cajanus Cajan*) (PDB ID: 4G7E) (Balasubramanian et al., 2013), and jack bean (*Canavalia ensiformis*) (PDB ID: 3LA4) (Balasubramanian et al., 2010). The conserved amino acids are colored in green. Certain conserved residues involved in the catalysis are indicated by asterisks. The active site flap for regulating the access of the substrates is highlighted by a blue dashed rectangle.

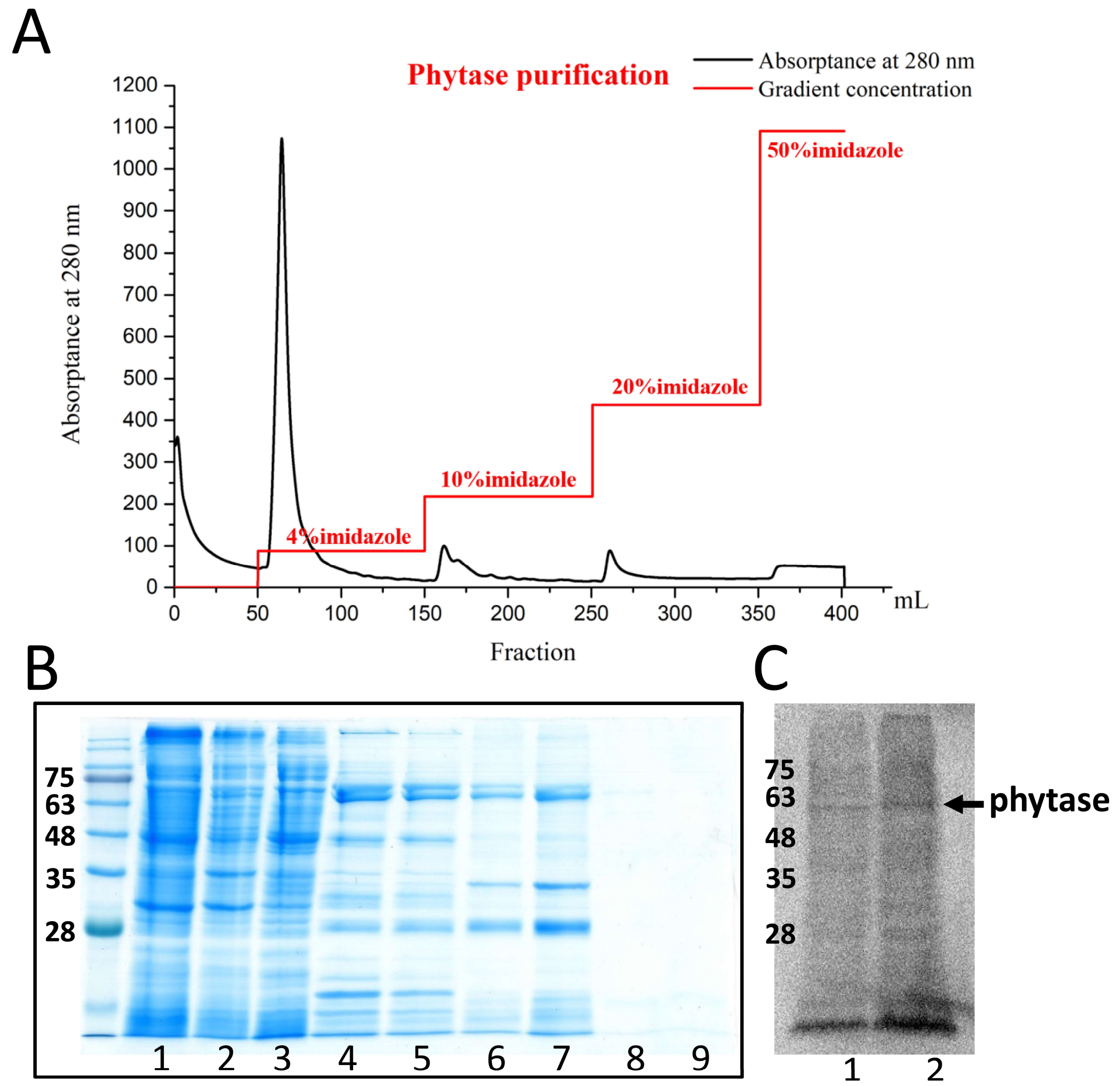

Fig. 7. SDS PAGE and western blot analysis of the expression of ASP1 phytase. (A) FPLC profile of the affinity purification, (B) with SDS-PAGE insert showing lane 4 and 5 – wash with 50 mM imidazole (10%); lane 6 and 7 – wash with 100 mM imidazole (20%); lane 8 and 9 - elution with 250 mM imidazole (50%). Lane 1 to 3, supernatant, pellet and flow through. (C) Western blot analysis: Arrow indicates expressed phytase in supernatant (lane 1) and pellet (lane 2).

References:

Balasubramanian, A., Durairajpandian, V., Elumalai, S., Mathivanan, N., Munirajan, A.,K., Ponnuraj, K. (2013) Structural and functional studies on urease from pigeon pea (*Cajanus cajan*). Int. J. Biol. Macromol. 58:301–309.

Balasubramanian, A., Ponnuraj, K. (2010) Crystal structure of the first plant urease from jack bean: 83 years of journey from its first crystal to molecular structure. J. Mol. Biol. 400:274–283.

Ha, N. C., Oh, B. C., Shin, S., Kim, H. J., Oh, T. K., Kim, Y. O., et al. (2000) Crystal structures of a novel, thermostable phytase in partially and fully calcium-loaded states. Nat. Struct. Biol. 7:147–153.

Lubkowski, J., Chan, W., Wlodawer, A. (2019) Opportunistic complexes of *E. coli* L-asparaginases with citrate anions. Sci. Rep. doi.org/10.1038/s41598-019-46432-0.

Swain, A. L., Jaskólski, M., Housset, D., Rao, J. K., Wlodawer, A. (1993) Crystal structure of *Escherichia colil*-asparaginase, an enzyme used in cancer therapy. Proc. Natl. Acad. Sci. USA. 90:1474–1478.

Yao, M., Yasutake, Y., Morita, H., Tanaka, I. (2005) Structure of the type I L-asparaginase from the hyperthermophilic archaeon *Pyrococcus horikoshii* at 2.16 angstroms resolution. Acta. crystallographica. Section D, Biological crystallography. 61:294–301.
